# SIRT2 and ALDH1A1 as critical enzymes for astrocytic GABA production in Alzheimer’s disease

**DOI:** 10.1186/s13024-024-00788-8

**Published:** 2025-01-15

**Authors:** Mridula Bhalla, Jinhyeong Joo, Daeun Kim, Jeong Im Shin, Yongmin Mason Park, Yeon Ha Ju, Uiyeol Park, Seonguk Yoo, Seung Jae Hyeon, Hyunbeom Lee, Junghee Lee, Hoon Ryu, C. Justin Lee

**Affiliations:** 1 Center for Cognition and Sociality, Life Science Institute (LSI), Institute for Basic Science (IBS), Daejeon, Republic of Korea; 2 IBS School, University of Science and Technology (UST), Daejeon, Republic of Korea; 3 College of Information and Biotechnology, Ulsan National Institute of Science and Technology (UNIST), Ulsan, Republic of Korea; 4 Division of Bio-Medical Science & Technology, Department of Neuroscience, KIST School, Korea University of Science and Technology, Seoul, Republic of Korea; 5 Brain Science Institute, Korea Institute of Science and Technology (KIST), Seoul, Republic of Korea; 6 Center for Advanced Biomolecular Recognition, Korea Institute of Science and Technology (KIST), Seoul, Republic of Korea; 7 Boston University Alzheimer’s Disease Research Center and Department of Neurology, Boston University Chobanian & Avedisian School of Medicine, Boston, USA; 8 VA Boston Healthcare System, Boston, USA; 9 KHU-KIST Department of Converging Science and Technology, Kyung Hee University, Seoul, 02447 Republic of Korea

**Keywords:** Alzheimer’s disease, Reactive astrocytes, SIRT2, GABA, Amyloid-beta, ALDH1A1

## Abstract

**Background:**

Alzheimer’s Disease (AD) is a neurodegenerative disease with drastically altered astrocytic metabolism. Astrocytic GABA and H_2_O_2_ are associated with memory impairment in AD and synthesized through the Monoamine Oxidase B (MAOB)-mediated multi-step degradation of putrescine. However, the enzymes downstream to MAOB in this pathway remain unidentified.

**Methods:**

Using transcriptomics analysis, we identified two candidate enzymes, Aldehyde Dehydrogenase 1 family member A1 (ALDH1A1) and Sirtuin 2 (SIRT2) for the steps following MAOB in the astrocytic GABA production pathway. We used immunostaining, metabolite analysis and electrophysiology, both in vitro and in vivo, to confirm the participation of these enzymes in astrocytic GABA production. We checked for the presence of SIRT2 in human AD patients as well as the mouse model APP/PS1 and finally, we selectively ablated SIRT2 in the astrocytes of APP/PS1 mice to observe its effects on pathology.

**Results:**

Immunostaining, metabolite analysis, and electrophysiology recapitulated the participation of ALDH1A1 and SIRT2 in GABA production. Inhibition of SIRT2 reduced the production of astrocytic GABA but not H_2_O_2_, a key molecule in neurodegeneration. Elevated expression of these enzymes was found in hippocampal astrocytes of AD patients and APP/PS1 mice. Astrocyte-specific gene-silencing of SIRT2 in APP/PS1 mice restored GABA production and partially improved memory function.

**Conclusions:**

Our study is the first to identify the specific role of SIRT2 in reactive astrogliosis and determine the specific pathway and metabolic step catalyzed by the enzyme. We determine the partial, yet significant role of ALDH1A1 in this process, thereby highlighting 2 new players the astrocytic GABA production pathway. Our findings therefore, offer SIRT2 as a new tool to segregate GABA from H_2_O_2_ production, aiding future research in neurodegenerative diseases.

**Graphical Abstract:**

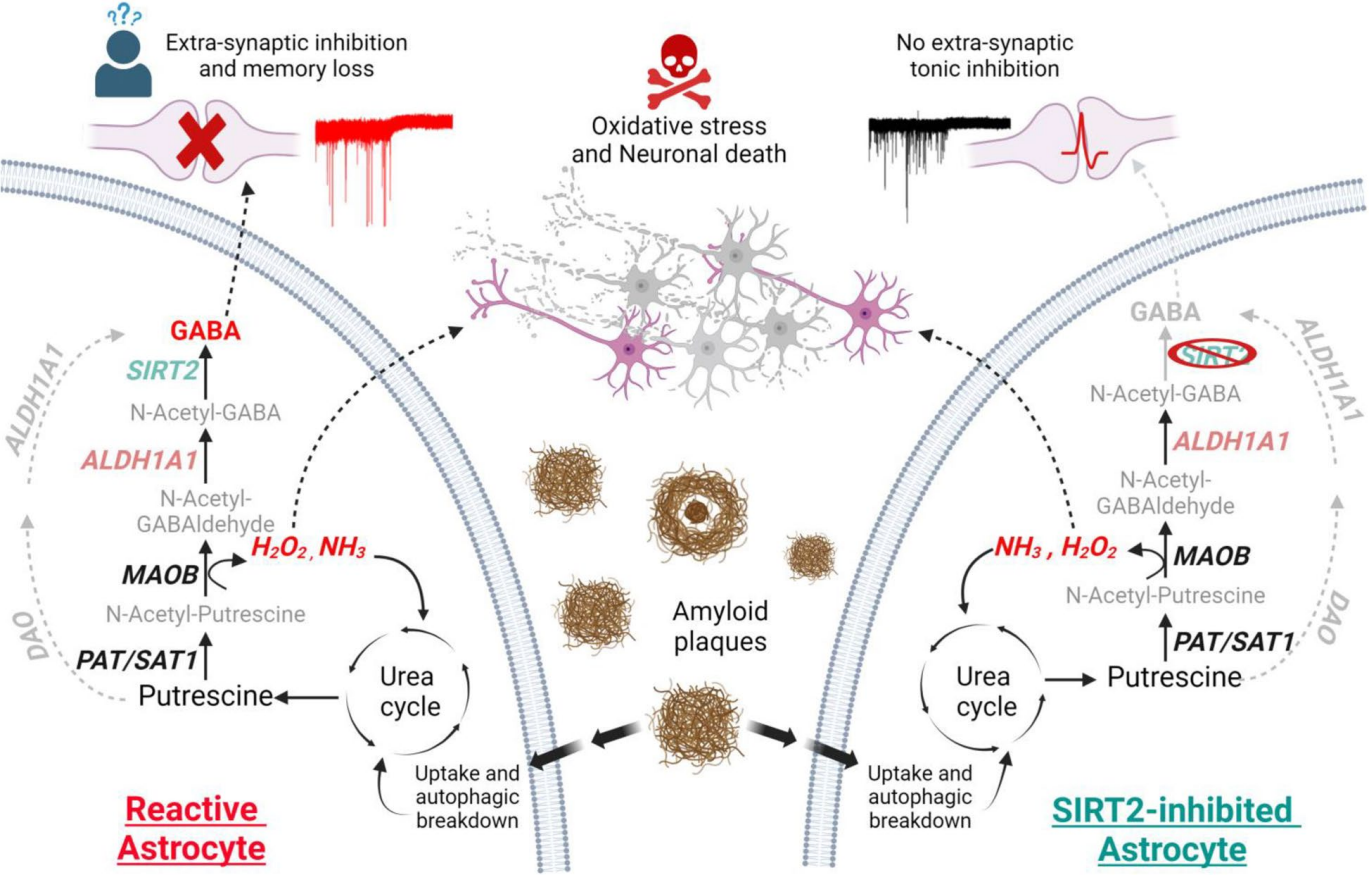

**Supplementary Information:**

The online version contains supplementary material available at 10.1186/s13024-024-00788-8.

## Background (Introduction)

Astrocytes play a myriad of roles in the brain, including but not limited to maintaining molecular and cellular homeostasis in the brain, regulating neuronal signaling and protecting against oxidative damage [1–3]. Alzheimer’s Disease (AD), a progressive and devastating neurodegenerative disorder characterized by neuroinflammation, amyloid-beta deposition, and neuronal death, features notable changes in astrocytic function and morphology, as initially observed by Alois Alzheimer, who referred to these cells as “glialzellen” [4]. Significant molecular and metabolic changes in reactive astrocytes in AD have been reported [5–7]. Among these changes, the production and tonic release of the inhibitory neurotransmitter γ-aminobutyric acid (GABA) by reactive astrocytes in neurodegenerative diseases has been highlighted recently [8–11]. However, previous studies and our current knowledge of GABA production in astrocytes only provide a partial picture about the molecular players involved in the process, particularly the participating enzymes and their regulatory roles. A comprehensive understanding of this process can help us realize therapeutic strategies against neurodegenerative disorders and target GABA production in a more cell-type-specific manner.

GABA in astrocytes can have two major sources: the GABA that is taken up from extracellular spaces by the GABA transporter, and the endogenous synthesis of GABA from precursors glutamate or putrescine [12]. Glutamic acid decarboxylase (GAD65 or GAD67)-mediated conversion of glutamate to GABA has been hypothesized but not been clearly determined in previous studies [13]. Putrescine has been shown to be converted to GABA via monoamine oxidase B (MAOB)-dependent degradation [14]. In addition to the MAOB-dependent pathway, the existence of an alternate diamine oxidase (DAO)-mediated conversion of putrescine to GABA has also been elucidated in thalamic astrocytes [15, 16].

**Fig. 1:**
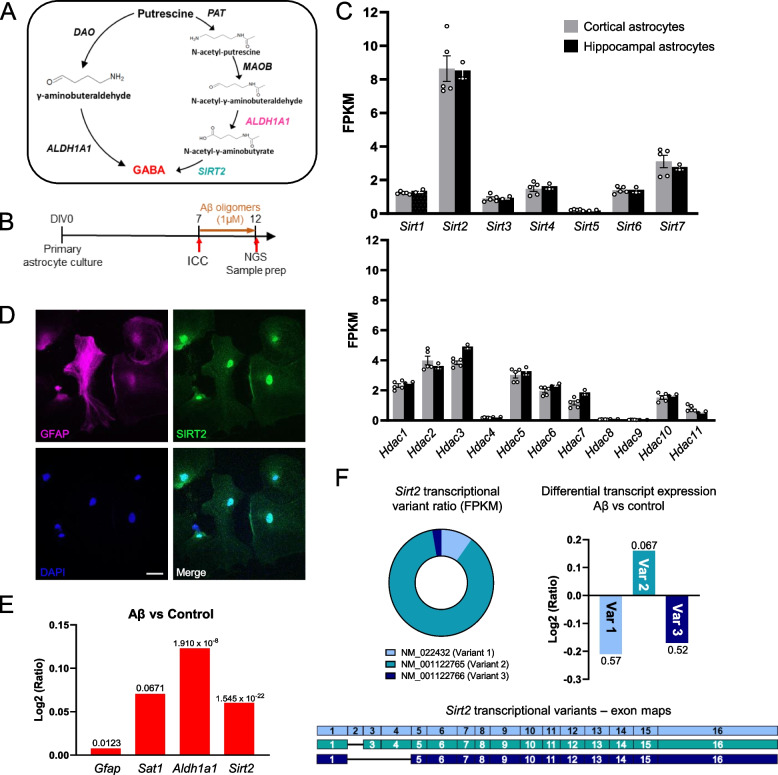
Sirt2 is highly expressed in cortical and hippocampal astrocytes. **A** Schematic diagram of the putrescine-to-GABA conversion pathways with predicted candidate enzymes. **B** Experimental timeline for immunocyctochemistry and Next Generation RNASeq. **C** Bar graph of FPKM values of sirtuin family members (top) and histone deacetylases (bottom) in primary cortical and hippocampal astrocyte cultures (individual data points refer to cell culture batches. **D** Immunostaining for SIRT2 and GFAP in primary astrocyte cultures (Scale bar 40 μm). **E** Differential expression analysis of genes using RNASeq in astrocytes upon Aβ treatment (Values above bars indicate FDR step-up from DeSeq2 differential analysis). **F** Top Left, pie chart representation of ratio of Sirt2 transcriptional variants in astrocytes; Top Right, bar graph of differential expression analysis of Sirt2 transcriptional variants (Values above bars indicate p-value from DeSeq2 differential analysis); Bottom, representation

MAOB-mediated conversion of putrescine to GABA is a 4-step pathway [14, 16], as opposed to the 2-step conversion mediated by DAO and Aldehyde Dehydrogenase 1 family member A1 (ALDH1A1) [15]. Putrescine is converted to N-acetyl-putrescine in the presence of Coenzyme A by enzyme putrescine acetyl transferase (PAT; also known as spermine/spermidine acetyl transferase SSAT1/SAT1) [10, 17], which is further oxidized to N-acetyl-γ-aminobutyraldehyde (N-acetyl-GABA) by MAOB [14]. The enzymes downstream to MAOB in this pathway have not been well studied, although their enzymatic functions can be determined based on the intermediate metabolites in the pathway (Fig. 1A). Following oxidation by MAOB, the intermediate aldehyde is further oxidized to N-acetyl-GABA by an aldehyde dehydrogenase (ALDH) family member, currently speculated to be ALDH2 [18], widely known for its role in alcohol metabolism in the liver [19]. Researchers have shown the involvement of ALDH2 in monoamine metabolism in mitochondrial extracts from rat livers [20] and in the metabolism of ethanol to produce GABA in cerebellar astrocytes [21], but there is no report of its involvement in the production of GABA from putrescine. However, among the 19 known ALDH family members, it has been reported that ALDH1A1 mediates the synthesis of GABA in midbrain dopaminergic neurons [22] and thalamic astrocytes [15], and is highly expressed in adult mouse hippocampal astrocytes [23], raising the possibility of its involvement in astrocytic MAOB-mediated conversion of putrescine to GABA as well.

Further, N-acetyl-GABA is deacetylated by an unknown deacetylase to finally synthesize GABA [24]. Major protein deacetylases in the cell can be classified as histone deacetylases (HDACs) or sirtuins (SIRTs). There is mounting evidence highlighting the role of sirtuins in several models of neurodegeneration. There are 7 proteins belonging to the SIRT family in humans, all of which have been implicated in neurodegenerative disorders [25]. Sirtuin proteins, although known to primarily be histone deacetylases, can also localize in non-neuronal subcellular regions, thereby participating in other metabolic processes. Among the 7 sirtuin proteins, the beneficial effect of SIRT2 inhibition in AD, Parkinson’s Disease and Huntington’s Disease [25] indicates its potential role in neurodegeneration and disease pathogenesis. We therefore hypothesize that SIRT2 participates in the deacetylation of N-acetyl-GABA to GABA in astrocytes.

In this study, we sought to determine the enzymes downstream to MAOB involved in the astrocytic putrescine-to-GABA conversion pathway. We hypothesized ALDH1A1 and sirtuins to be potential candidates for this, due to their notable expression levels and participation in astrocytic GABA-associated disease pathology. To investigate our hypothesis, we performed Next Generation RNA-sequencing (NGS) to detect the expression of different deacetylases in astrocytes and changes in their levels in AD-like conditions. In cultured astrocytes, we measured the changes in putrescine-induced GABA and N-acetyl-GABA levels on pharmacological and genetic manipulation of SIRT2 activity. Further, we measured the production and release of GABA from hippocampal astrocytes in culture as well as ex vivo on pharmacological and genetic manipulation of ALDH1A1 or SIRT2 to confirm our findings. In the brains of AD-like mouse model APP/PS1 as well as human AD patients, we observed increased expression of astrocytic SIRT2. Additionally, cell-type-specific gene silencing of SIRT2 in the astrocytes was able to restore the aberrant GABA production and memory deficits in APP/PS1 mice. Indeed, we were able to demonstrate the role of SIRT2 and ALDH1A1 in the putrescine-induced production of GABA in astrocytes.

## Methods

### Animal husbandry

Experiments were performed either on C57BL/6 (B6) animals acquired from the Institute for Basic Science Animal Facility (larf.ibs.re.kr) or on AD mouse model mouse APPswe/PSEN1dE9 (APP/PS1) mice of B6C3 hybrid background (RRID: MMRRC_034829-JAX), originated from Jackson Laboratory (USA, stock number 004462) and maintained as hemizygotes by crossing transgene-carrying mice with B6C3 F1 animals. Genotype of the APP/PS1 animals were determined by PCR using the following primers—APP/PS1_F—5’ AAT AGA GAA CGG CAG GAG CA 3’; APP/PS1_R—5’ GCC ATG AGG GCA CTA ATC AT 3’. All mice used in this study were maintained in a 12-h light/dark cycle (lights out at 2000 h) and were allowed *ad libitum* access to food and water. Animals were maintained in accordance with the guidelines stated by the Institutional Animal Care and Use Committee of IBS (Daejeon, South Korea). B6 littermates at 8-weeks of age were used for virus injection experiments, followed by slice patch clamp recordings at 14 weeks old. Virus injection and subsequent slice patch, behavioral tests and IHC were performed on 12–16 month old APP/PS1 animals and age-matched wild type (WT) littermates.

### Primary astrocyte culture

Primary astrocytes were cultured from P1 pups of C57BL/6 J mice as previously described [26]. Briefly, the cerebral cortex and hippocampus were dissected and cleaned of meninges and midbrain before dissociation into a single cell suspension by trituration in astrocyte culture medium. The medium was prepared by using Dulbeccos’ modified Eagle’s Medium (DMEM, Corning) supplemented with 4.5 g/L glucose, L-glutamine, sodium pyruvate, 10% heat-inactivated horse serum, 10% heat-inactivated fetal bovine serum and 1000 units/mL of penicillin–streptomycin. Cells were plated onto culture dishes coated with 0.1 mg/mL poly-D-lysine (Sigma) and maintained in astrocyte culture medium at 37 °C in a humidified atmosphere containing 5% CO_2_. Three days later (at DIV4), cells were vigorously washed with Dulbecco’s phosphate buffered saline by repeated pipetting and the media was replaced.

### Illumina Hiseq library preparation and RNA sequencing

RNA was isolated from cultured primary astrocytes using Qiagen RNEasy Kit (Qiagen, #74,104). Sample libraries were prepared using the Ultra RNA Library Prepkit (NEBNext, #E7530), Multiplex Oligos for Illumina (NEBNext, #E7335) and polyA mRNA magnetic isolation module (Invitrogen, #61,011) following manufacturers’ instructions. Full details of the library preparation and sequencing protocol are provided on the website and previously described [27]. The Agilent Bioanalyser and associated High Sensitivity DNA Kit (Agilent Technologies) were used to determine the quality, concentration, and average fragment length of the libraries. The sample libraries were prepared for sequencing according to the HiSeq Reagent Kit Preparation Guide (Illumina, San Diego, CA, USA). Briefly, the libraries were combined and diluted to 2 nM, denatured using 0.1N NaOH, diluted to 20 pM by addition of Illumina HT1 buffer and loaded into the machine along with read 1, read 2 and index sequencing primers. After the 2 × 100 bp (225 cycles) Illumina HiSeq paired-end sequencing run was complete, the data were base called and reads with the same index barcode were collected and assigned to the corresponding sample on the instrument, which generated FASTQ files for analysis.

### NGS Data analysis

BCL files obtained from Illumina HiSeq2500 were converted to fastq and demultiplexed based on the index primer sequences. The data was imported to Partek Genomics Suite (Flow ver 10.0.21.0328; copyright 2009, Partek, St Louis, MO, USA), where the reads were further processed. Read quality was checked for the samples using FastQC. High quality reads were aligned to the *Mus musculus* (mouse) genome assembly GRCm38 (mm10, NCBI) using STAR (2.7.3a). Aligned reads were quantified to the mouse genome assembly (mm10, RefSeq transcripts 93) and normalized to obtain fragments per kilobase million (or FPKM) values of positively detected and quantified genes. Gene read counts were also normalized to Transcripts per million (TPM), which was used to identify alternate splice variants of the positively detected genes. Differential gene analysis was carried out by normalizing the quantified and annotated gene reads to the Median Ratio and performing DeSeq2 (available on Partek Genomics Suite).

### cDNA synthesis and qRT-PCR

cDNA was synthesized from 500 μg RNA as previously described, using Superscript III Reverse Transcriptase (Enzynomics, #RT006M). 20 ng cDNA (per reaction tube) was used to perform qRT-PCR using Power SYBR Green PCR Master Mix (Applied Biosystems, #4,367,659) to check the expression level of SIRT2 and compare against GAPDH (primers used listed below).

Mouse *Gapdh* qRT-F: ACC CAG AAG ACT GTG GAT GG

Mouse *Gapdh* qRT-R: CAC ATT GGG GGT AGG AAC AC

Mouse *SIRT2* qRT-F: TCA TCA GCA AGG CAC CAC TA

Mouse *SIRT2* qRT-R: GTC CCT GTA AGC CTT CTT GG

### Immunocytochemistry

For pharmacological study, astrocytes (DIV 7–10) were seeded on coverslips and incubated with 180 μM putrescine (Sigma-Aldrich, P5780) in the presence or absence of 10 μM DEAB, 200 nM EX527 or 3 μM AGK2 overnight. For genetic ablation study, DIV 7 astrocytes were detached from culture dish surface, electroporated with mCherry-tagged pSicoR vector carrying Scr sequence or shRNA sequences against SIRT2 or ALDH1A1 (shSIRT2 targeting 5’-GGAGCATGCCAACATAGATGC-3’ or shALDH1A1 targeting 5’-TTTCCCACCATTGAGTGCC-3’ respectively) and seeded onto coverslips. Two days later, they were treated with putrescine (180 μM) for 24 h. Cells on the coverslips were fixed with 4% paraformaldehyde (PFA; Sigma-Aldrich) in 0.1 M PBS at room temperature for 15 min. After fixation, the coverslips were washed 3 times with 0.1 M PBS for 10 min each, then blocked with 0.1 M phosphate-buffered saline (PBS) containing 0.3% Triton X-100 (Sigma, USA) and 10% Donkey Serum (Genetex) for 1.5 h at room temperature. The cells were then incubated with primary antibodies in a blocking solution in the following composition: guinea-pig anti-GABA antibody (1:500, AB175, Millipore), chicken anti-GFAP antibody (1:1000, AB5541, Millipore), rabbit-anti-SIRT2 (1:200, ab211033, abcam) for overnight (16 h or more) at 4 °C with gentle rocking. After washing 3 times with 0.1 M PBS, 10 min each, the cells were incubated with corresponding secondary antibodies in blocking solution in the following composition: conjugated Alexa 647 donkey-anti-chicken anti IgG (1:500, 703–605-155, Jackson), Alexa 488 donkey anti-guinea-pig IgG (1:500, 706–545-148, Jackson), Alexa 488 donkey-anti-rabbit IgG (1:200, 711–547-003, Jackson) for 2 h at room temperature with gentle rocking. The cells were then incubated with 1:2000 DAPI solution (Pierce) in 0.1 M PBS for 10 min followed by 3 rinses with 0.1 M PBS. Coverslips were finally mounted onto slide glass with fluorescence mounting solution (S3023, DAKO, USA). Images were acquired using a Nikon A1R confocal microscope (pharmacological study) or Zeiss LSM900 microscope (genetic ablation study) and analyzed using the ImageJ program (NIH).

### 2-cell sniffer patch clamp recording

Primary astrocyte cultures were prepared from P1 C57BL/6 mouse pups as described above. As required, the cells were seeded onto poly-D-lysine-coated cover glass and either electroporated with respective shRNA constructs (genetic ablation experiments) or treated with inhibitors in the presence of putrescine (pharmacological inhibition) on DIV7. On the day of sniffer patch, HEK 293-T cells expressing GFP-tagged GABA_C_ receptors were seeded onto the astrocytes and allowed to settle for at least 1 h before patching. The cover glasses were then immersed in 5 μM Fura-2-AM (in 1 mL external HEPES solution containing 5μL of 20% pluronic acid) for 40 min to allow Fura incorporation into the cell, washed at room temperature (with external HEPES solution, described later) and subsequently transferred to the microscope stage. The external HEPES solution of the following composition (in mM): 150 NaCl, 10 HEPES, 3 KCl, 2 CaCl_2_, 2 MgCl_2_, 5.5 glucose (pH adjusted to 7.3, osmolality to 320 mOsmol kg^−1^) was allowed to continuously flow over the cells during the experiment and was replaced with one containing 100 μM GABA during full activation recording. Images at 510 nm wavelength were taken after excitation by 340 nm and 380 nm light using pE-340^fura^ (CoolLED) to record calcium transients within the cells. The two resulting images were used for ratio calculations in Axon Imaging Workbench (version 11.3, Axon Instruments). To perform the sniffer patch, the astrocytic TRPA1 receptor was activated by pressure poking with a glass pipette and the resulting GABA release was recorded as inward current in the GABA_C_-GFP-expressing HEK 293 T cells under voltage clamp (V_h_ = −60 mV) using Axopatch 200A amplifier (Axon Instruments), acquired with pClamp 11.3. Recording electrodes (4–10 MΩ) were filled with the following internal solution (in mM): 110 Cs-gluconate, 30 CsCl, 0.5 CaCl_2_, 10 HEPES, 4 Mg-ATP, 0.3 Na3-GTP and 10 BAPTA (pH adjusted to 7.3 with CsOH, osmolality adjusted to 300 mOsm kg^−1^ with sucrose). For the simultaneous recording of calcium response with the patch and poking pipettes, Imaging Workbench was synchronized with pClamp 11.3. To normalize for the differences in GABA_C_ receptor expression on the HEK cells, a saturating concentration of 100 μM GABA (in HEPES solution) was applied to record maximal GABA current from the cell, and the poking-induced current was normalized as a percentage of full activation current on application of GABA from the HEK cell.

### Metabolite analysis

Metabolite analysis for putrescine, acetyl-GABA, and GABA was performed using electrospray ionization UPLC-MS/MS system. The system used for the analyses was an Exion LC™ AD UPLC coupled with a Triple Quad 4500 MS/MS System (AB Sciex LLC, Framingham, USA) using an Acquity® UPLC BEH HILIC column (1.7 μm particle size, 2.1 mm x 100 mm, Waters, USA) at 30 °C, controlled by Analyst 1.6.2 software (AB Sciex LP, Ontario, Canada). Into the astrocyte sample pellets, 70% methanol (100μL) was added and was vortexed for 30 s. Three freeze/thaw cycles with liquid nitrogen were used to lyse cells, followed by a 10 min centrifugation at 20,817 g (14,000 rpm). DNA normalization was performed using 5μL of each sample's supernatant. For sample normalization, DNA concentrations in each sample were analyzed using a Nano-MD UV–Vis spectrophotometer (Scinco, Seoul, Korea). The internal standard (d_2_-GABA at a final concentration of 4 μM) was added to 20μL of the supernatant from each sample, and the mixture was vortexed for 30 s. Then the mixture was evaporated with gentle stream of nitrogen to dryness at 37℃ using a TurboVap evaporator (Biotage, Uppsala, Sweden). The residue was reconstituted by vortexing for 30 s and sonicating for 15 min using 25μL of the mobile phase A (0.1% formic acid in acetonitrile):B (50 mM ammonium formate, pH 4) = 8:2 solvent. The initial chromatographic conditions were at 80% solvent A at a flow rate of 0.4 mL/min. After 7 min at 80% A, solvent A was set to 5% over the next 0.5 min, and these conditions were retained for an additional 1 min. The system was then returned to the initial conditions over the next 0.5 min. The system was then left for 1.5 min in the initial conditions for re-equilibration. The total sample running time was 10.5 min. All samples were placed in the auto-sampler, maintained at 7 °C during the analysis, and the injection volume was 5μL. The analysis was performed using positive ESI mode. The ion spray voltage and vaporizer temperature were 5.5 kV and 380℃, respectively. The curtain gas was kept at 35 psi, and the collision gas was maintained at 8 psi. The nebulizer gas was 60psi, while the turbo gas flow rate was 70psi. The metabolites were detected selectively using their unique multiple reaction monitoring (MRM) pairs. The MRM parameter (Q1/Q3) for putrescine, acetyl-GABA, and GABA are (89.062 / 72.1), (146.011 / 86.015), and (103.979 / 87.1), respectively, to monitor specific parent-to-product transitions. The standard calibration curve for each metabolite was used for absolute quantification.

### Live-cell imaging of oROS-G in primary cultured mouse astrocytes

On DIV7, primary cultured astrocytes were transfected with AAV5-GFAP-oROS-GFP viral vector and seeded into a glass-bottom 96-well plate (ibidi; #89,627). 2 days later, cells were treated with putrescine (180 μM) in the presence or absence of enzyme inhibitors (AGK2, 3 μM or KDS2010, 1 μM) or vehicle and confocal live-cell imaging was performed using a Nikon A1R confocal microscope mounted onto a Nikon Eclipse Ti body with 20X objective lens. The plate was housed in a live-cell imaging chamber and maintained at 10% CO_2_ and 37 °C for 47 h of continuous recording. Images were acquired and analysed using the NIS-element AR (Nikon).

### Virus injection

Mice were anesthetized using isoflurane and head-fixed onto stereotaxic frames (Kopf). The scalp was incised, and a hole was drilled into the skull above the hippocampus (A/P −1.8, D/V −1.9 from bregma, M/L ± 1.2 from the skull surface). Viruses were loaded into a stainless steel needle in a 1:1 ratio (lentivirus:AAV) and injected bilaterally into the dentate gyrus at the rate of 0.1μL/min for 10 min (1μL in each hemisphere). Viruses used: AAV-GFAP-Cre-mCherry, lenti-pSico-Scr/shSIRT2/shALDH1A1-eGFP, were generated at the Institute for Basic Science Virus Facility (https://www.ibs.re.kr/virusfacility/). Mice were used 2–3 weeks after injection for behavior experiments and 6 weeks after injection for electrophysiological recordings.

### Acute brain slicing

Brain slices were prepped from 14–15 weeks old B6 animals or 12–16 months old transgenic (and WT littermate) APP/PS1 animals. Briefly, animals were deeply anaesthetized using isoflurane and the brain was swiftly excised from the skull in ice-cold high-sucrose artificial cerebrospinal fluid (aCSF) with the following composition (in mM): 212.5 sucrose, 26 NaHCO_3_, 10 d-( +)-glucose, 5 MgCl_2_, 3 KCl, 0.1 CaCl_2_, 1.25 NaH_2_PO_4_. Coronal slices of the hippocampus of 300 μm thickness were prepared and incubated at room temperature in extracellular aCSF for at least 1 h before recording. The extracellular aCSF solution used for incubation and recordings was composed as follows (in mM): 124 NaCl, 3 KCl, 24 NaHCO_3_, 2 CaCl, 1.25 NaH_2_PO_4_, 1 MgCl_2_, 10 d-( +)-glucose (osmolarity adjusted to 310 mOsm kg^−1^, pH 7.4). All solutions were maintained with constant bubbling with a mixture of 95%O_2_ and 5%CO_2_.

### Tonic GABA recording

Electrophysiological recordings were performed by placing the slices in a recording chamber continuously perfused with extracellular aCSF (flow rate 0.2 mL/min), mounted on the stage of an upright Olympus microscope and viewed with a 4X and a 60X water-immersible objective lens (0.90 numerical aperture) with infrared DIC optics. The cells were visualized using a charged-couple device camera and the Imaging Workbench software (INDEC Biosystems). Whole-cell patch clamp recordings were made from the granule cells located in the dentate gyrus, held at a potential of −70 mV. Patch pipettes (5-9MΩ) were filled with an internal solution of the following composition (in mM): 135 CsCl, 4 NaCl, 0.5 mM CaCl_2_, 10 HEPES, 5 EGTA, 10 QX-314, 2 Mg-ATP and 0.5 Na_2_-GTP. Once whole-cell configuration was obtained, baseline current was stabilized using d-AP5 (50 mM, Tocris) and 6-cyano-7-nitroquinoxaline-2,3-dione (20 mM; Tocris) after which aCSF additionally containing bicuculline (100 μM; Tocris) was flowed through the recording chamber to record the tonic GABA current. Electrical signals were digitized and samples at 50 μs intervals with Digidata 1440A and a Multiclamp 700B amplifier (Molecular Devices) using pCLAMP10.2 software. The amplitude of the tonic GABA current was measured as the baseline shift after bicuculline application using the Clampfit program.

### Mouse brain tissue sectioning and immunohistochemistry

Animals were anaesthetized using isoflurane and perfused with 0.9% saline, followed by ice-cold 4% PFA in 0.1 M PBS. The brain was excised and stored in 4% PFA at 4 °C overnight for post-fixation, followed by dehydrolysation in 30% sucrose for 48 h. Coronal sections of 30 μm thickness were prepared in a cryostat and stored in a glycerol-based storage solution at 4 °C till use. Before staining, the slices were washed in 0.1 M PBS thrice and incubated for 1 h in blocking solution (4% Donkey Serum, 0.3% Triton X-100 in 0.1 M PBS). Primary antibodies were added to the blocking solution at desired dilution and incubated overnight at 4 °C with gentle rocking. Unbound antibodies were washed off by rocking and rinsing the slices thrice with 0.1 M PBS, followed by 2 h of incubation at room temperature with corresponding fluorescence-tagged secondary antibodies (diluted in blocking solution). Unbound secondary antibodies were washed by rocking and rinsing thrice with 0.1 M PBS, the first wash of which contained 1:1000 DAPI for nuclear visualization. The slices were then mounted using a fluorescence mounting medium (Dako) and dried.

Antibodies used in the experiments were as follows (dilutions in blocking solution)—rabbit-anti-ALDH1A1 (1:200, ab52492, abcam), chicken-anti-GFAP (1:500; AB5541, Millipore), rabbit-anti-SIRT2 (1:200, ab211033, abcam), Alexa 647 donkey-anti-chicken anti IgG (1:500, 703–605-155, Jackson), Alexa 488 donkey-anti-rabbit IgG (1:200, 711–547-003, Jackson), PyrPeg (1 μM, incubated along with secondary antibodies). 22–24 μm Z-stacked images in 2 μm steps were processed using the ZEN Digital Imaging for Light Microscopy blue system (Zeiss, ver. 3.2) and ImageJ (NIH, ver. 1.54b) software.

### Mouse behavior tests

Behavior experiments were performed 2–3 weeks after virus injection. All test subjects were handled for 10 min by the experimenter every day for 5 days before behavior recording at the same time during the day/night cycle in the same room as the behavior setup. Behavior experiments were carried out in the light phase circadian cycle.

For the Open Field test, animals were placed in a 40 cm × 40 cm chamber with no markings and allowed to explore uninterrupted for 10 min. They were tracked for the last 8 min of the recording (Ethovision XT, Noldus) to measure the total distance travelled and the velocity.

On the day of the Y-maze experiment, the test animals were put in the middle of a symmetrical Y maze with 3 identical arms (30 cm long x 15 cm high) and allowed to explore freely for 10 min. The animal was recorded and the last 8 min of the videos were studied by an experimenter, blinded to the animal test condition, to analyze the total number of arm entries and the alternation behavior. Data was analyzed to exclude any abnormal behavior (one test subject was removed from the study due to reduced mobility). An entry was considered only when all 4 limbs of the animal were within the arm. The percentage of alternation was calculated as follows:

$$Percent\;alternation=\lbrack(total\;number\;of\;alternations)/(total\;number\;of\;arm\ entries-2)\rbrack\ast100.$$

For the Novel Place Recognition test, animals were placed into a 40 cm x 40 cm chamber with one marked wall and 2 identical objects placed close to the marked wall (see schematic in Fig. S2 for reference). Animals were allowed to roam freely and explore the objects for 8 min before returning to their home cage. 60 min later, one object was moved further away from the marked wall and the animal was returned to the chamber. Animal exploration of each object was tracked for 8 min. Recorded videos were analyzed by an experimenter blinded to animal condition. Exploring was considered when the animal sniffed at the object without attempting to climb it in the recorded video. Ratio of preference (Ret./Acq.) was calculated as follows:

$$\begin{array}{c}Preference\;in\;Acq\;=\;(Time\;spent\;exploring\;object-to-be-moved)/Total\;exploration\;time\\Preference\;in\;Ret\;=\;(Time\;spent\;exploring\;moved\;object)/Total\;exploration\;time\\Ratio=(Preference\;in\;Retention)/(Preference\;in\;Acquisition)\end{array}$$

### Western blot

The hippocampus was dissected from the acute brain slices after patch clamp recordings were done and immediately frozen. Protein was extracted from the samples by homogenization in RIPA buffer (Rockland; MB-030–0050) containing 100 × Phosphatase Inhibitor (GenDEPOT; P320-001) and estimated using the BCA assay (Thermo Scientific; #23255). 20 μg of protein was prepared (boiling with marker), loaded into a gradient 4–15% SDS protein gel (BioRAD; #4561084) and then separated via electrophoresis. The separated proteins were transferred onto a PVDF membrane (Invitrogen; B24002) and blocked for 1 h at room temperature with 5% skim milk (Difco) in TBST solution. The membrane was then rinsed thrice with TBST and incubated with primary antibodies in 3% BSA (GenDEPOT; A0100-010) in TBST and sodium azide overnight at 4 °C. Membranes were subsequently washed thrice with TBST and incubated with appropriate HRP-conjugated antibodies (in 5% skim milk in TBST) for 1 h at room temperature. Membranes were thoroughly washed with TBST (3–4 times) and the HRP was visualized using ECL Western Blotting Substrate (Thermo Scientific; #32106). Expression (or knockdown) of SIRT2 was calculated relative to the protein loading control β-actin. Antibodies used – 1:1000 Rabbit anti-SIRT2 (abcam; ab211033); 1:2000 Rabbit anti-beta actin (Cell Signaling; 490L); 1:3000 Goat-anti-Rabbit HRP (Sera Care; 5450–0010).

### Human brain samples

Neuropathological examination of postmortem brain samples from normal subjects and AD patients was determined using procedures previously established by the Boston University Alzheimer’s Disease Center (BUADC). Demographic information of humans used in this study is provided in Table S1. Next of kin provided informed consent for participation and brain donation. Institutional review board approval for ethical permission was obtained through the BUADC center. This study was reviewed by the Institutional Review Board of the Boston University School of Medicine and was approved for exemption because it only included tissues collected from post-mortem subjects not classified as human subjects. The study was performed in accordance with institutional regulatory guidelines and principles of human subject protection in the Declaration of Helsinki.

### Double chromogenic staining for human brain tissues

Sequential double staining was performed using horse radish peroxidase (HRP) and alkaline phosphatase (AP) substrates as chromogens for two different antigens, respectively.

#### First staining

Paraffin-embedded tissues were sectioned in a coronal plane at 10 μm. BLOXALL® Blocking solution (Vector Laboratories, Burlingame, CA, USA) was used to block endogenous alkaline phosphatase. Tissue sections were blocked with 2.5% normal horse serum (Vector Laboratories) for 1 h and then incubated with GFAP antibody (1:400; AB5541, Millipore) for 24 h. After reaction with secondary antibodies, tissue slides were processed with Vector ABC Kit (Vector Laboratories). The GFAP immunoreactive signals were developed with DAB chromogen (Thermo Fisher Scientific).

#### Second staining

Tissue slides stained with GFAP were incubated with SIRT2 antibody (1:200; ab211023, abcam) for 24 h to confirm localization of SIRT2 in reactive astrocytes. After washing three times with PBS, sections were incubated with ImmPRESS-AP anti-rabbit IgG (alkaline phosphatase) polymer detection reagent (Vector Laboratories: MP-5401) for 2 h at room temperature. A Vector Red alkaline phosphatase substrate kit (Vector Laboratories: SK-5105) was used to develop SIRT2 signals. Hematoxylin (Vector Laboratories: H-3401–500) was added to tissue slides to visualize the nuclei of the cells. Double-stained tissue slides were gradually processed back to xylene through an increasing ethanol gradient [70%, 80%, 90%, 95%, and 100% (1 time)], and subsequently mounted. The chromogenic signals of GFAP (brown) and SIRT2 (red) were examined under a light microscopy (BX63; Olympus, Japan) equipped with high definition (1920 × 1200 pixel) digital camera (DP74; Olympus).

### Quantification and statistical analysis

All analyses were done blindly. The statistical tests used in each figure are listed in the legends. Numbers and individual dots refer to individual samples (or individual cells) unless mentioned otherwise in figure legends. N represents the number of animals used for the experiment. Data representation and statistical analysis was performed using GraphPad Prism (Graphpad Software). For image analysis, ImageJ (NIH) and Nikon A1R (Nikon) were used. Statistical significance was set at ∗*p* < 0.05, ∗∗*p* < 0.01, ∗∗∗*p* < 0.001 and ∗∗∗∗*p* < 0.0001 (unless mentioned otherwise in figure legends).

## Results

### Next generation sequencing reveals high expression of SIRT2 in primary cultured astrocytes

To begin, we examined the putrescine-to-GABA conversion pathway in the brain and dissected the molecular processes involved in each step (Fig. 1A). To investigate the presence and abundance of deacetylases in astrocytes, we performed Next Generation RNA-Sequencing (NGS) to objectively compare the expression levels of our candidate deacetylases in primary astrocyte cultures derived from the cortex as well as hippocampus of P1 mice (Fig. 1B, C). We screened the FPKM levels of different Sirtuin genes and histone deacetylases and found that, among these genes, Sirtuin 2 (*Sirt2*) had the highest expression level in both mouse cortical as well as hippocampal astrocytes (Fig. 1C). Although SIRT2 is majorly known for its role in microtubule deacetylation, the protein can also translocate to the nucleus to modulate the cell cycle [28]. Using immunocytochemistry, we determined the localization of the SIRT2 protein in the cytoplasm as well as the nucleus of primary cultured mouse hippocampal astrocytes (Fig. 1D), indicating that it can actively participate in cellular metabolic processes in astrocytes. We found that 5-day treatment of oligomerized amyloid beta (Aβ 1 μM; abcam), which has been known to induce AD-like astrocyte reactivity in vitro [27] upregulated the expression of candidate enzymes Putrescine Acetyltransferase (*Sat1*), SIRT2 (*Sirt2*) and ALDH1A1 (*Aldh1a1*) involved in the putrescine-to-GABA degradation pathway (Fig. 1E) in hippocampal astrocytes. Furthermore, transcriptional variant analysis showed that transcriptional variant 2 of mouse *Sirt2* (NM_001122765.2), which is the majorly expressed variant in the central nervous system that translates to a functional protein [29], was specifically upregulated (Fig. 1F), altogether suggesting that SIRT2 was a viable candidate for our proposed deacetylase enzyme.

### SIRT2, not SIRT1, and ALDH1A1 are involved in the conversion of putrescine to GABA in primary cultured astrocytes

**Fig. 2:**
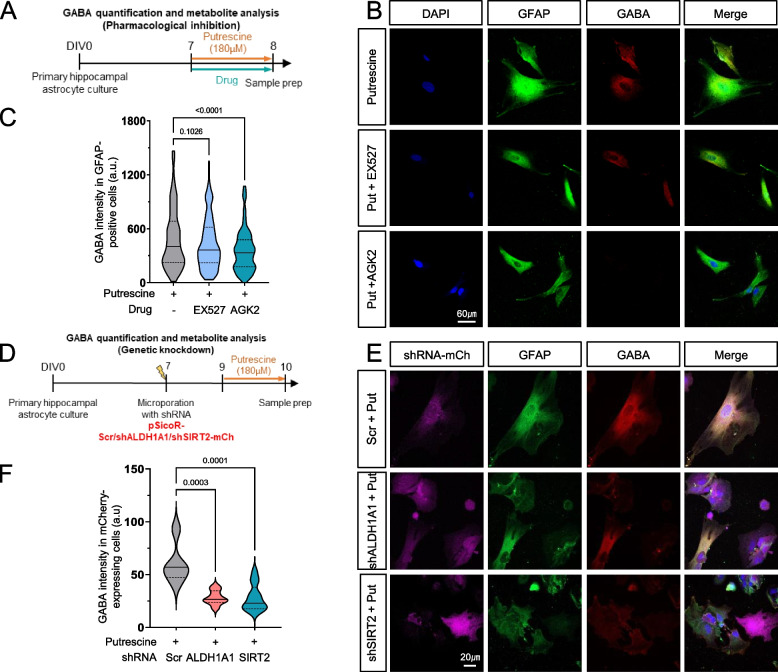
SIRT2 and ALDH1A1 are involved in the conversion of putrescine to GABA in astrocytes (See also Fig. S1). **A** and **D** Experimental timeline for GABA staining and quantification with pharmacological (**A**) and genetic (**D**) inhibition. **B** and **E** Pseudo-colored representative images from immunostaining for GFAP and GABA in primary cultured astrocytes treated with putrescine in the presence or absence of EX527 or AGK2 (**B**) or putrescine-treated primary cultured astrocytes expressing Scr/shALDH1A1/shSIRT2-mCherry (**E**). **C** and **F** Truncated violin plot for GABA intensity in GFAP-positive cells from (**B**), and mCherry-positive (shRNA-expressing) cells from (**E**). Data represents Median ± quartiles. Values above graphs represent p-value from indicated statistical test (Ordinary one-way ANOVA)

To examine GABA production by putrescine treatment in cultured astrocytes and confirm the role of SIRT2 in the process, we treated mouse hippocampal astrocyte cultures with putrescine (180 μM) in the absence or presence of inhibitors for SIRT1 and SIRT2, EX527 (200 nM) and AGK2 (3 μM) [30] respectively, for one day and performed immunostaining against GFAP and GABA (Fig. 2A-C). While EX527 treatment had no effect on putrescine-induced GABA levels in the cells, we saw a significant reduction in the accumulation of GABA in the cells treated with SIRT2 inhibitor AGK2 (Fig. 2C), confirming that SIRT2 was involved in the GABA-production pathway. To further rule out the non-specific inhibition of other deacetylases by AGK2, we synthesized a short hairpin RNA (shRNA) sequence specific to *Sirt2* and transfected hippocampal astrocytes to genetically knockdown the expression of SIRT2 (Fig. S1, Fig. 2D-F). Additionally, to verify the involvement of ALDH1A1 in GABA production, we electroporated hippocampal astrocytes with shRNA targeting *Aldh1a1* [15] and checked GABA levels after 1 day of putrescine treatment (Fig. 2E) by immunochemistry. As a result, putrescine-induced GABA levels were reduced in primary cultured hippocampal astrocytes upon genetic ablation of *Aldh1a1* as well as *Sirt2* (Fig. 2F), suggesting their role in putrescine-to-GABA conversion in astrocytes. Taken together, we propose that ALDH1A1 and SIRT2 are the unknown enzymes downstream of MAOB in the astrocytic GABA production pathway.

### SIRT2 inhibition reduces GABA production while accumulating upstream metabolites

**Fig. 3:**
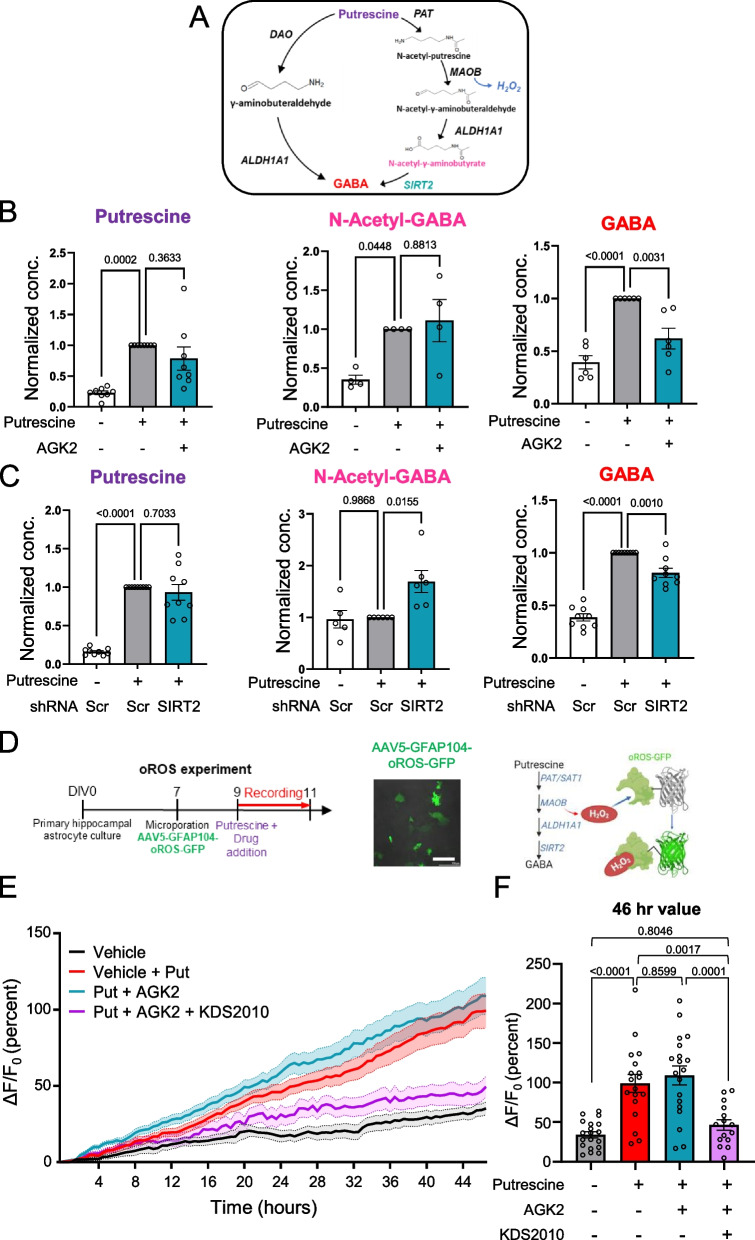
SIRT2 inhibition reduces GABA production while accumulating N-Acetyl-GABA and H_2_O_2_ in astrocytes. **A** Pathway schematic showing putrescine-to-GABA metabolism. **B** Bar graphs for relative metabolite concentration in primary astrocyte cultures treated with or without putrescine, in the presence or absence of AGK2 (normalized to metabolite concentration in putrescine-treated cells). **C** Bar graphs for relative metabolite concentration in primary cultured astrocytes expressing Scr/shSIRT2-mCherry treated with or without putrescine (normalized to metabolite concentration in putrescine-treated Scr-mCh-expressing cells). **D** Experimental timeline for the oROS experiments (Left), representative fluorescent image showing GFP-tagged oROS sensor expression in cultured astrocytes (Middle; Scale bar 250 μm), schematic illustration of putrescine-induced H_2_O_2_ and its detection by the oROS sensor (Right). **E** Average ΔF/F_o_ (percent) traces obtained from 46-h live-cell monitoring of putrescine-induced H_2_O_2_ production in various conditions. **F** Bar graph representing average ΔF/F_o_ value at 46 h (individual data points represent single ROIs evaluated, Ordinary one-way ANOVA). Data represents Mean ± SEM. Values above graphs represent p-value from corresponding statistical test. **B** and **C** Individual data points represent different cell culture vessels (RM one-way ANOVA)

To investigate the direct changes in the intermediates formed during the conversion of putrescine to GABA, we performed liquid chromatography-mass spectrometry (LC–MS) analysis in primary cultured hippocampal astrocytes after 1-day treatment of putrescine in the presence and absence of SIRT2 inhibitor AGK2 (Fig. 3). We found that intracellular levels of putrescine and SIRT2 substrate N-acetyl GABA were 2- to threefold higher in putrescine treated cells and remained unchanged on SIRT2 inhibition (Fig. 3B). Consistent with our previous findings (Fig. 2C), GABA levels increased on putrescine treatment, which were brought down to control levels upon inhibition of SIRT2. We also corroborated our results by genetically ablating SIRT2 via shRNA (Fig. 3C). While the data for putrescine levels was consistent with our pharmacological inhibition experiments, it was noteworthy that we observed over 1.5-fold accumulation of N-Acetyl-GABA upon knockdown of SIRT2 in putrescine-treated astrocytes, indicating that SIRT2 was specifically involved in the metabolic conversion of N-acetyl-GABA to GABA (see Fig. 3A). Intracellular GABA levels were similarly decreased by SIRT2 inhibition (Fig. 3C, Right). These results indicate that SIRT2 is a key enzyme in the production of GABA from putrescine, via the deacetylation of the intermediate metabolite N-Acetyl-GABA.

We propose SIRT2 to be the enzyme involved in the last step of the astrocytic putrescine-to-GABA conversion pathway (Fig. 3A). Since the LC/MS analysis revealed that genetic inhibition of SIRT2 caused accumulation of substrate N-acetyl-GABA (Fig. 3C), we hypothesized that the pathway upstream to this last step continued functioning unhindered, thereby producing hydrogen peroxide (H_2_O_2_) during the conversion of N-acetyl-putrescine to N-acetyl-γ-aminobutyraldehyde by MAOB [31, 32]. To corroborate this, we microporated a genetically encoded ROS sensor oROS-G into primary cultured hippocampal astrocytes and performed live-cell imaging for 47 h, to observe the temporal changes in ROS levels within the cells (Fig. 3D). The binding of peroxide molecule to this novel green fluorescent sensor induces the formation of a disulfide bridge that results in fluorescence that can be detected using a confocal microscope [33], the intensity of which acts as a measure of cellular H_2_O_2_ levels. As a result, we observed that H_2_O_2_ levels increased in putrescine-treated astrocytes as compared to control (vehicle-treated) cells, which remained unchanged upon SIRT2 inhibition by AGK2 (Fig. 3E and F). Peroxide levels in the astrocytes were significantly reduced upon the inhibition of MAOB by KDS2010 (1 μM) [16]. Taken together, we confirmed that SIRT2 was involved in the production of GABA from putrescine, specifically in the last step of the metabolic process, and that inhibition of SIRT2 reduces GABA production while allowing continuous production of H_2_O_2_ from upstream enzymatic reactions.

### SIRT2 is essential, while ALDH1A1 is partially responsible for GABA production from putrescine in astrocytes

**Fig. 4:**
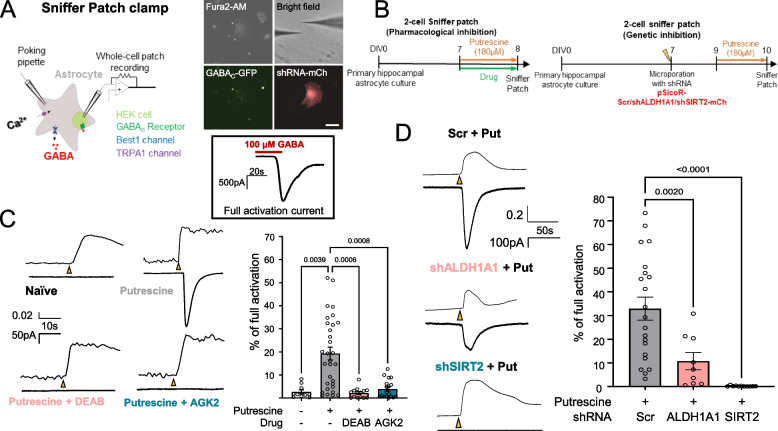
SIRT2 is essential, while ALDH1A1 is partially involved in GABA production. **A** Schematic for working of 2-cell sniffer patch experiments (Left) Representative fluorescence images of the sniffer patch experiment (Right top, scale bar 40 μm) and representative trace of full activation of GABA_C_-expressing HEK cells by GABA treatment (Right bottom). **B** Experimental timeline for 2-cell sniffer patch experiments with pharmacological inhibition (Left) or genetic ablation (Right) of target enzymes. **C** Representative traces (Left) and bar graph (Right) of GABA release-mediated sensor current from astrocytes treated with or without putrescine in the presence or absence of DEAB or AGK2. **D** Representative traces (Left) and bar graph (Right) of GABA release-mediated sensor current from putrescine-treated cultured astrocytes expressing Scr/shALDH1A1/shSIRT2-mCherry. Data represents Mean ± SEM. Individual data points represent each cell recorded. Values above graphs represent p-value from corresponding statistical test (Ordinary One-way ANOVA)

To further examine the release of the GABA produced on accumulation of excess putrescine in astrocyte cultures, we performed 2-cell sniffer patch experiments on putrescine-treated astrocytes in the presence or absence of appropriate inhibitors or genetic inhibition (Fig. 4A and B). Briefly, on poking the cell membrane, astrocytic TRPA1 channels are activated and cause an influx of calcium ions into the cell, which can be recorded as a calcium signal using fura-2-AM [34]. This calcium influx causes GABA release from the astrocyte via the BEST1 channel [35], which can be measured as current recorded on a nearby GFP-tagged GABA_C_ receptor-expressing HEK293T cell (Fig. 4A) and can be used as a measure of intracellular GABA level in the poked astrocyte. One-day treatment of putrescine significantly increased the amount of GABA released from the astrocyte (Fig. 4C), which was significantly eliminated by the inhibition of ALDH1A1, using DEAB (N,N-diethylaminobenzaldehyde, 1 μM), or SIRT2, using AGK2 (3 μM) (Fig. 4C). Since DEAB is not specific to ALDH1A1 and also inhibits other members of the ALDH-family of proteins [36], we validated our results by using shRNA specific to *Aldh1a1* (Fig. 4D). Interestingly, we found that while DEAB was able to eliminate the putrescine-induced GABA current when compared to vehicle treatment, shALDH1A1 was only able to eliminate 65% of the poking-induced GABA release (Fig. 4D), implying the role of other DEAB-sensitive aldehyde dehydrogenase enzymes in the conversion of putrescine to GABA. Genetic ablation of SIRT2 using shRNA was able to eliminate poking-induced GABA release from astrocytes (Fig. 4D). Taken together, our results indicate a partial role of ALDH1A1 and key role of SIRT2 in the production of GABA from putrescine in astrocytes.

**Fig. 5:**
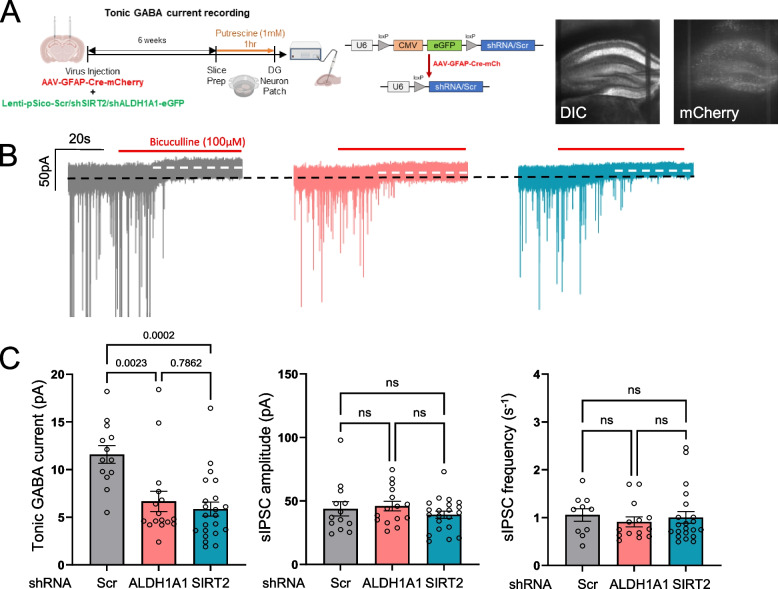
SIRT2 and ALDH1A1 are involved in GABA production in vivo. **A** Schematic of virus injection and experimental timeline for tonic GABA recordings in B6 mice (Left); Virus constructs used for genetic knockdown of target enzymes (Middle); Representative DIC and fluorescence images of virus infection and spread (Right). **B** Representative traces of tonic GABA current recorded in the DG neurons from putrescine-incubated brain slices of AAV-GFAP-Cre + Lenti-pSico-Scr/shSIRT2/shALDH1A1-GFP-injected B6 mice. **C** Representative bar graphs for tonic GABA current (Left), spontaneous IPSC amplitude (Middle) and frequency (Right) from recorded traces. Data represents Mean ± SEM. Data points represent individual cells recorded from N = 5 in each group. Values above graphs represent p-value from corresponding statistical test (Ordinary One-way ANOVA, Tukey’s multiple comparisons)

To validate our findings in vivo, we induced astrocyte-specific knockdown of SIRT2 or ALDH1A1 in the hippocampus of 8-week old B6 mice and performed whole-cell patch clamp recordings in the dentate gyrus (DG) after incubation with putrescine (1 mM) for 1 h [17] (Fig. 5). Lentiviruses carrying constructs pSico-Scr/shSIRT2/shALDH1A1-eGFP each were injected in the DG along with AAV-GFAP-Cre-mCherry-carrying virus (Fig. 5A) to knockdown SIRT2 or ALDH1A1 in an astrocyte-specific manner. 6 weeks after injection, we measured the tonic GABA current from the DG and observed that 1 h of putrescine incubation at 37 °C was sufficient to induce GABA production and release from the astrocytes in the Scr-injected animals (Fig. 5B and C). Astrocytic knockdown of ALDH1A1 or SIRT2 reduced the production and tonic release of GABA from the astrocytes, as measured from the DG granule layer neurons (Fig. 5B and C). Neuronal GABA production and synaptic release was not affected by the knockdown of these genes, as evidenced by the spontaneous IPSC amplitude and frequency recorded from these cells (Fig. 5C middle, right). Since the targeted genes were knocked down only in astrocytes that successfully expressed the AAV-GFAP-Cre-mCh construct, the reduction in tonic GABA levels was not as absolute as observed in the in vitro sniffer patch experiment (Fig. 4D). These results demonstrate that astrocytic SIRT2 and ALDH1A1 are necessary for the production of putrescine-induced GABA in hippocampal astrocytes.

### SIRT2 and ALDH1A1 are elevated in APP/PS1 animals and contribute to aberrant tonic GABA and memory deficits

**Fig. 6:**
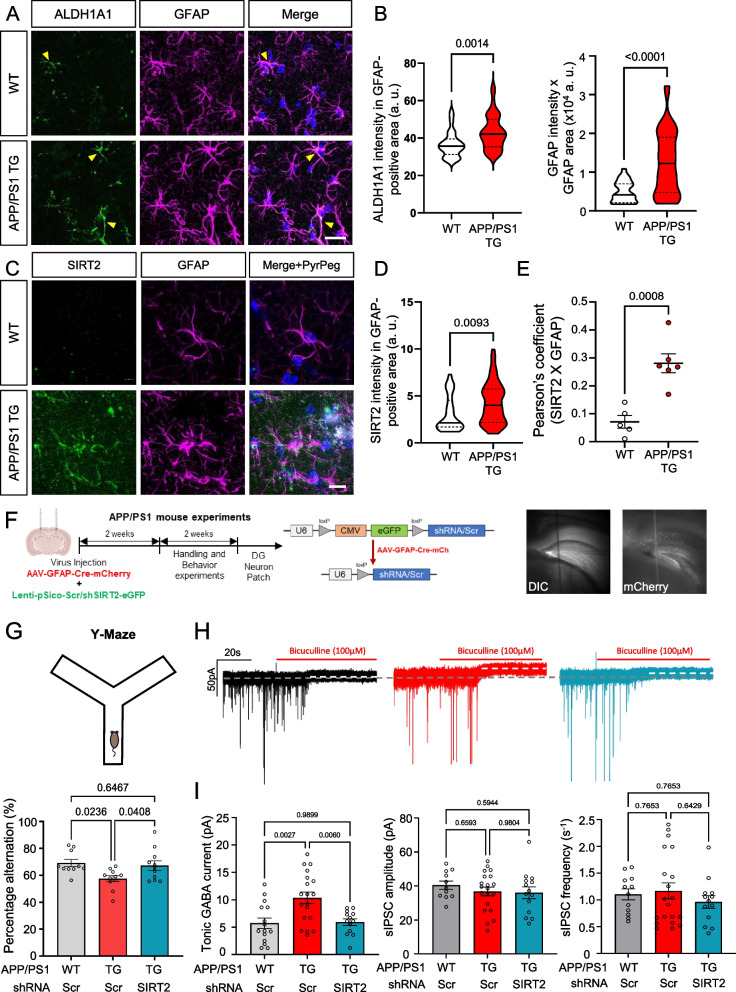
SIRT2 and ALDH1A1 are involved in Alzheimer’s Disease pathology (See also Fig. S2). **A** Images representing ALDH1A1 and GFAP immunoreactivity in the CA1 *stratum radiatum* in the hippocampus of APP/PS1 mice (Scale bar 20 μm). **B** Violin plot representing the astrocytic ALDH1A1 intensity (Left) and GFAP immunoreactivity (Right) in APP/PS1 mouse hippocampus (*N* = 3, Mann–Whitney test). **C** Lattice SIM images of SIRT2 immunostaining and localization with GFAP immunoreactivity near amyloid beta plaques (Stained with PyrPeg, white) in APP/PS1 mice (Scale bar 10 μm). **D** Violin plot representing SIRT2 intensity in GFAP-positive astrocytes in APP/PS1 mice (*N* = 3, Mann–Whitney test). **E** Dot-plot representation of Pearson’s coefficient for colocalization of SIRT2 and GFAP in Lattice SIM images from (**C**) (individual data point represents one image, *N* = 3; Unpaired t-test). **F** Schematic representation of animal experimental timeline (Left), virus constructs for cell type-specific genetic knockdown (Middle) and representative DIC and fluorescence images showing virus expression and spread (Right). **G** Y-maze schematic diagram (Top) and bar graph representing spontaneous alternation (in percentage) of APP/PS1 mice in Y-maze behavior task (Bottom; individual data point represents one animal; Ordinary one-way ANOVA). **H** Representative traces of tonic GABA current recorded from DG granule layer neurons of APP/PS1 mice from (**G**). **I** Bar graphs representing the tonic GABA current (Left), spontaneous IPSC amplitude (Middle) and frequency (Right) from recorded traces (individual data point represents one cell recorded, *N* = 4–6 each group; Ordinary one-way ANOVA). Data represents Median ± quartiles or Mean ± SEM. Values above graphs refer to p values from statistical analyses

We next examined the levels of ALDH1A1 and SIRT2 proteins in the astrocytes of APP/PS1 animals, a mouse model that is frequently used to study AD [8] (Fig. 6). We performed immunohistochemistry using antibodies against GFAP, ALDH1A1 and SIRT2 in the hippocampus of wild-type (WT) and transgenic (TG) APP/PS1 animals (Fig. 6A to E). We found that ALDH1A1 was expressed significantly higher in the hippocampal astrocytes of APP/PS1 animals as compared to their WT littermates (Fig. 6A, B). We further confirmed astrocytic reactivity by measuring the GFAP-positive area and intensity and observed a significant elevation in APP/PS1 astrocytic area and GFAP expression (Fig. 6B, right).

Further, we performed immunostaining for SIRT2 in the astrocytes surrounding amyloid beta plaques using PyrPeg, a molecule that can selectively detect neuritic plaques [37] and performing super-resolution microscopy (Fig. 6C). We observed an obvious lack of PyrPeg as well as SIRT2 immunoreactivity in WT animals, while the expression of SIRT2 in hippocampal astrocytes was significantly higher around the Aβ plaques in APP/PS1 animals (Fig. 6D). Since SIRT2 is known to be expressed in non-astrocytic cell types of the brain as well [38], we analyzed the colocalization of SIRT2 and GFAP in the microscopic images. The Pearson’s coefficient was significantly increased in APP/PS1 animal brains (Fig. 6E), suggesting an astrocytic role of the protein in disease conditions. These results implicated a pathological relevance of SIRT2 in the astrocytes of AD.

In order to determine the role of SIRT2 in aberrant GABA production in AD, we performed astrocytic knockdown experiments in APP/PS1 animals by injecting lentiviruses carrying pSico-Scr/shSIRT2-eGFP each and AAV-GFAP-Cre-mCherry virus in the hippocampal DG (Fig. 6F, Fig. S2). Two weeks after injection, we observed that spontaneous alternation in Y-maze, which is used as a measure of working spatial memory, was reduced in Scr-injected APP/PS1 animals, compared to WT, and this deficit was rescued by astrocytic SIRT2 knockdown (Fig. 6G), without affecting the animal mobility (Fig. S2). Additionally, whole-cell patch clamp of DG granule layer neurons revealed elevated tonic GABA currents in Scr-injected APP/PS1 animals (Fig. 6H and I), consistent with previous reports [8, 27], which was rescued down to WT-levels by astrocytic knockdown of SIRT2. We confirmed knockdown of SIRT2 in the hippocampus using western blot (Fig. S2B, C). Taken together, we demonstrate the role of SIRT2 in aberrant GABA production from reactive astrocytes by genetically knocking it down and rescuing tonic GABA current as well as memory deficits.

### SIRT2 expression is elevated in the hippocampus of AD patients

**Fig. 7:**
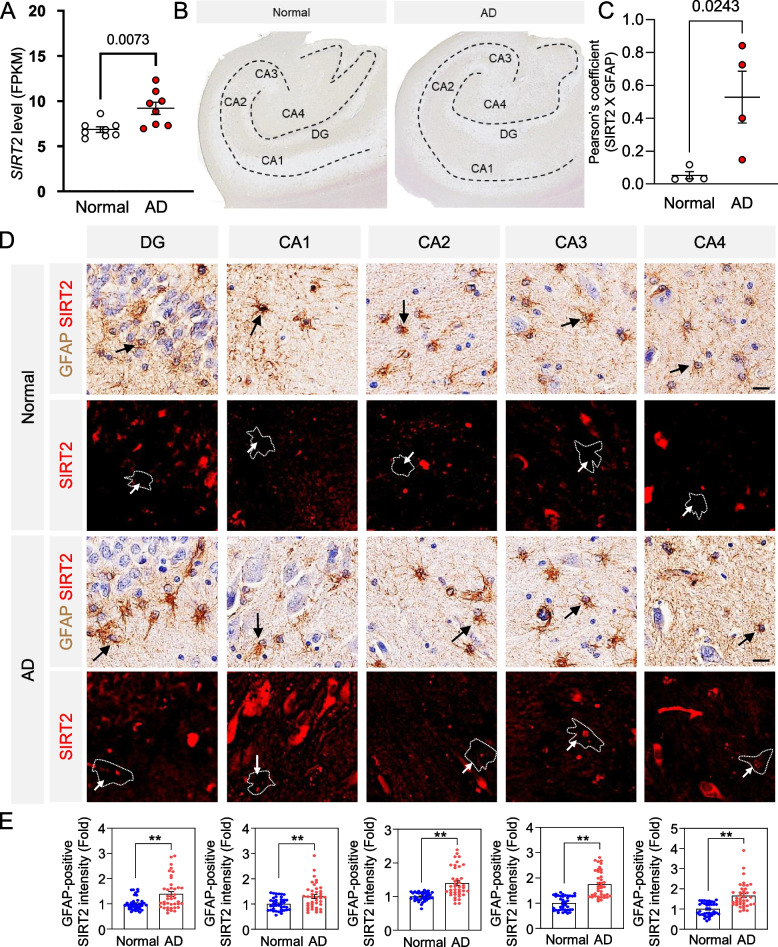
SIRT2 immunoreactivity is increased in GFAP-positive astrocytes of the hippocampus of AD patients. **A** Dot-plot representing average FPKM value obtained from transcriptomics of human post-mortem brain tissue. **B** Representative image of the hippocampal section from normal subject and AD patient. **C** Dot-plot representing Pearson’s coefficient of SIRT2 x GFAP colocalization in images from (**D**) (individual dot refers to one image). **D** Representative images of SIRT2 and GFAP immunoreactivity in the hippocampal regions (DG, CA1, CA2, CA3, and CA4) of human brain tissue. Black arrows indicate GFAP-positive astrocytes (brown), and white arrows indicate SIRT2 signals (red). **E** Densitometry analysis of SIRT2 immunoreactivity in GFAP-positive area from images in (**D**) of the corresponding hippocampal regions (individual dots represent ROIs corresponding to individual cell). Data represents Mean ± SEM. Data recorded from 4 samples in each condition, 10 cells per sample (Unpaired t-test)

To assess the clinical significance of SIRT2 in humans, we obtained transcriptome data from post-mortem human AD patient brains and compared *SIRT2* expression levels against healthy individuals (Fig. 7A). We found that the mRNA level for *SIRT2* was significantly higher in AD patient brain tissue as compared to healthy individuals. We further performed immunohistochemistry to confirm the astrocytic localization of SIRT2 and found significant elevation of SIRT2 intensity in the astrocytes (GFAP-positive area) of the hippocampal CA1, CA2, CA3, CA4, and DG in AD patients (Fig. 7 B, D and E). We confirmed the astrocyte cytoplasmic presence of the protein by checking Pearson’s coefficient of colocalization of SIRT2 and GFAP (Fig. 7C and D black and white arrows). We, therefore, propose that astrocytic SIRT2 has a clinically significant role in AD in the production of astrocytic GABA from accumulated putrescine.

## Discussion

In this study, we have delineated the enzymes involved in the MAOB-mediated metabolism of putrescine to GABA in hippocampal astrocytes. Based on RNA-Seq data analysis, we identified SIRT2 as the best candidate for the final deacetylation step (Fig. 1) and demonstrated its role by pharmacological inhibition (AGK2) and gene silencing (shSIRT2) in vitro as well as ex vivo. Inhibition or genetic ablation of SIRT2 leads to reduced GABA production in primary cultured astrocytes on immunostaining (Fig. 2), metabolite analysis (Fig. 3) and 2-cell sniffer patch (Fig. 4). Furthermore, SIRT2 inhibition also causes accumulation of predicted substrate N-acetyl-GABA as well as H_2_O_2_ (Fig. 3), which are upstream to SIRT2, supporting our hypothesis. We reveal that ALDH1A1 also participates in astrocytic putrescine-to-GABA conversion (Figs. 2, 4 and 5). Based on our findings, we propose that ALDH1A1 and SIRT2 are the enzymes downstream to MAOB in astrocytic GABA production pathway (Fig. 1A). These findings are further confirmed by the elevated expression of SIRT2 in human AD patients as well as in APP/PS1 mouse brains, indicating its role in disease pathology. Our study unveils that cell-type-specific genetic ablation of SIRT2 in the hippocampal astrocytes of APP/PS1 mice was able to reverse disease pathology, providing us with another unexplored and unique target to battle the devastating disease.

Our study provides the first line of evidence that SIRT2 is involved in GABA production in astrocytes. SIRT2 is majorly known for its role in the cell cycle via α-tubulin deacetylation [38] and H4K16 deacetylation [39], and it has been reported that the levels of the protein increase as cells senesce [28]. While it has previously been demonstrated that the inhibition of SIRT2 reduces astrocyte reactivity markers [40] and rescues α-synuclein-mediated toxicity in Parkinson’s Disease models [30], our study is the first to identify the metabolic step catalyzed by SIRT2 in reactive astrocytes. The accumulation of intermediate metabolite N-acetyl-GABA in primary cultured astrocytes on genetic ablation of the enzyme as well as the uninterrupted H_2_O_2_ production on SIRT2 inhibition (Fig. 3) implicates that SIRT2 catalyzes this final acetyl-transfer step. This must, however, be validated by testing the NAD + dependency of this process, as SIRT2 is an NAD + -dependent deacetylase.

SIRT2 has been known to have different functions in different cell types. In oligodendrocyte precursor cells, it is known to be involved in proliferation and differentiation [38]. Inhibition of SIRT2 in cervical cancer cells proves to have a beneficial effect by causing cell cycle arrest [41]. SIRT2 is also known to be involved in the critical pentose phosphate pathway [42] and subsequently in maintaining the redox balance of the cell via Glutathione peroxidase deacetylation [43]. These multifacted and delicate functions of SIRT2 caution us against proposing SIRT2 as a therapeutic target against AD. Interestingly, the SIRT2 KO animals show elevated rates of tumorigenesis, altered locomotion and memory deficits, implicating a delicate role of the protein in cell cycle regulation and brain function [44]. Our discovery of the involvement of SIRT2 in astrocytic GABA production points to yet another critical role in brain function that was previously unexplored, possibly in the modulation of excitation-inhibition balance via GABA production. This exciting possibility awaits future study.

While ALDH2 has been reported to be involved in monoamine metabolism in the liver [20] and alcohol metabolism in the brain [21], our NGS data reported upregulation of *Aldh1a1* in Aβ-treated AD-like astrocytes (Fig. 1), suggesting the involvement of ALDH-family members other than ALDH2 in this process. Putrescine-induced GABA production was inhibited by DEAB (Fig. 4), which is reported to have little, if any, inhibitory effect on ALDH2 [36], further supporting that ALDH2 does not participate in this process. While DEAB was able to largely eliminate putrescine-induced GABA in cultured astrocytes (Fig. 4C), genetic knockdown of ALDH1A1 only showed a 65% reduction in GABA release (Fig. 4D). As DEAB is well-known to inhibit several enzymes from the family [36], we predict other ALDH-family members that use DEAB as a substrate could be involved in this oxidation step. There also exists the possibility that another ALDH enzyme is switched-on in compensation for the genetic knockdown in Fig. 4, and this possibility awaits further study. While other ALDH-family members may be involved in this step, we were able to determine that ALDH1A1 plays the major role of aldehyde dehydrogenation in GABA production and therefore, can be studied in the future in the context of therapeutic applications.

Astrocytes are known as the main defense against oxidative species in the brain [45] and therefore, changes in their reactive state can be critical in pathologies, such as AD, PD and even aging [46]. Accumulation of oxidative species in astrocytes in a reactive state can cause mitochondrial dysfunction and further oxidative damage, thereby creating a cascading loop for degeneration in the brain, where they are unable to scavenge ROS to maintain homeostasis while also adding more ROS to the system. The by-products H_2_O_2_ and ammonia during the MAOB-mediated production of GABA are key molecules causing reactive astrogliosis in neurodegenerative diseases [27, 31]. The inhibition of SIRT2, downstream to MAOB, does not reduce the production of these molecules (Fig. 3). This allows us to segregate aberrant astrocytic GABA and H_2_O_2_ production to precisely determine the cause of neurodegeneration and memory deficits in AD. Results from our experiments (Fig. 6) demonstrate that rescuing hippocampal astrocytic GABA production in APP/PS1 mice is sufficient to rescue memory deficits. However, this rescue effect was limited and could not be seen in other behavioral tests, such as the Novel Place Recognition test for spatial memory (Fig. S2). The partial memory rescue can be explained by the lack of neuronal death observed in APP/PS1 animals [47], possibly due to lesser ROS production compared to the newly developed GiD model for AD, which reflects a significant neuronal loss in the hippocampus [31]. Upon revealing the existence of SIRT2 downstream to the H_2_O_2_-producing step in GABA metabolism, we can now reverse aberrant GABA production (Figs. 5 and 6) while continually producing H_2_O_2_ in the system (Fig. 3). Our study, therefore, not only identifies the previously unexplored role of SIRT2 in astrogliosis but also provides a tool to dissect the effects of harmful molecules GABA and H_2_O_2_ produced by astrocytes in disease pathogenesis.

Reactive astrocytes in AD mediate neuroinflammation [48] and have been implicated indirectly in Aβ deposition [49], which results in severe changes in cellular functions such as energy metabolism [50], acetate metabolism [51], autophagic clearance of amyloid plaques [52], and more recently discovered, the urea cycle and the downstream MAOB-mediated putrescine-to-GABA pathway [17, 27]. Together with the results from our previous studies [8, 17, 27] we present AD as a metabolism-altering disease, adding to the current literature on the subject [5–7, 50]. The newly developed tools and concepts from this study should be useful in understanding the metabolic switch of astrocytes from resting to reactive state as the underlying cause of pathology and neurodegeneration in AD.

## Supplementary Information

Supplementary Material 1: Table S1: Information of postmortem brain tissues from normal subjects and AD patients. Table S2: Detailed information for statistical analysis. 

Supplementary Material 2: Figure S1: shRNA construct design and testing for shSIRT2. Figure S2: Astrocyte-specific knockdown of SIRT2 has partial effect on memory rescue in APP/PS1 mice.

## Data Availability

The RNASeq data used in this study will uploaded onto NCBI GEO repository at the time of publication. The datasets used and/or analysed during the current study are available from the corresponding author on reasonable request. Raw images obtained from microscopy can be provided by the corresponding author upon request.

## Abbreviations

Aβ: Amyloid beta
AD: Alzheimer’s Disease
ALDH: Aldehyde Dehydrogenase
ALDH1A1: Aldehyde Dehydrogenase 1 family member A1
BSA: Bovine Serum Albumin
DAO: Diamine Oxidase
DEAB: N,N-diethylaminobenzaldehyde
DG: Dentate gyrus
FPKM: Fragments per kilobase per million mapped fragments
GABA: γ—Aminobutyric acid
GFAP: Glial Fibrillary Acid Protein
HDAC: Histone Deacetylase
HRP: Horseradish Peroxidase
IHC: Immunohistochemistry
LC/MS: Liquid Chromatography/Mass Spectrometry
MAOB: Monoamine Oxidase B
NGS: Next Generation Sequencing
PBS: Phosphate Buffered Saline
ROS: Reactive Oxygen Species
shRNA: Short hairpin RNA
SIRT: Sirtuin
TBST: Tris buffered Saline – Tween20
TG: Transgenic
WT: Wild type
