# Supplementary material for: SIRT2 and ALDH1A1 as critical enzymes for astrocytic GABA production in Alzheimer’s disease
Source: Mol Neurodegener. 2025 Jan 15;20:6. doi: 10.1186/s13024-024-00788-8 (PMC11734448; doi:10.1186/s13024-024-00788-8)
Supplement: Supplementary file 1 — Supplementary Material 1: Table S1: Information of postmortem brain tissues from normal subjects and AD patients. Table S2: Detailed information for statistical analysis. [file 13024_2024_788_MOESM1_ESM.docx]

**SIRT2 and ALDH1A1 as critical enzymes for astrocytic GABA production in Alzheimer’s Disease**

Mridula Bhalla^1,2^, Jinhyeong Joo^1,2^, Daeun Kim^1,3^, Jeong Im Shin^1,4,5^, Yongmin Mason Park^1,2^, Yeon Ha Ju^6^, Uiyeol Park^6^, Seonguk Yoo^7^, Seung Jae Hyeon^6^, Junghee Lee^8,9^, Hyunbeom Lee^7^, Hoon Ryu^6^*, C Justin Lee^1,2^ *

**Supplementary Information**

**Table S1 (Related to Fig. 7)** Information of postmortem brain tissues from normal subjects and AD patients.

**Table S2.** Detailed information for statistical analysis

**Table S1 (Related to Fig. 7)**

**Information of postmortem brain tissues from normal subjects and AD patients.**

| Number | Case | Age | Sex | Braak stage |
| --- | --- | --- | --- | --- |
| 1 | Normal | 88 | M | I |
| 2 | Normal | 82 | M | I |
| 3 | Normal | 70 | M | I |
| 4 | Normal | 87 | F | I |
| 5 | Normal | 83 | F | I |
| 1 | AD | 89 | M | V |
| 2 | AD | 83 | M | VI |
| 3 | AD | 70 | M | VI |
| 4 | AD | 84 | F | VI |
| 5 | AD | 88 | F | VI |

**Table S2. Detailed information for statistical analysis**

| **Figure No.** | **Result from statistical analysis** |
| --- | --- |
| 2C | Vehicle (487.9 ± 23.87, *n*=194), EX527 (434.7 ± 18.97, *n*=224), AGK2 (356.5 ± 16.30, *n*=202)  Ordinary one-way ANOVA (Dunnett’s multiple comparisons test) F(2, 617) = 10.69, *p* <0.0001  Vehicle vs EX527, *p =* 0.1026; Vehicle vs AGK2, *p* <0.0001. |
| 2F | Scr + Put (60.34 ± 8.629, *n*=5), shALDH1A1 + Put (28.13 ± 2.388, *n*=8), shSIRT2 + Put (28.06 ± 3.432, *n*=13)  Ordinary one-way ANOVA (Dunnett’s multiple comparisons test) F(2, 23) = 13.25, *p* =0.0001  Scr+Put vs shALDH1A1+Put, *p =* 0.0003; Scr+Put vs shSIRT2+Put, *p =* 0.0001. |
| 3B | **Putrescine**  Naïve (0.2282 ± 0.03038, *n*=8), Putrescine (1.000 ± 0.000, *n*=8), AGK2 (0.7842 ± 0.1875, *n*=8).  Ordinary one-way ANOVA (Tukey’s multiple comparisons test) F(2, 21) = 13.18, *p* =0.0002  Naïve vs Putrescine, *p =* 0.0002; Putrescine vs AGK2, *p =* 0.3633. |
|  | **N-Acetyl-GABA**  Naïve (0.3509 ± 0.05755, *n*=4), Putrescine (1.000 ± 0.000, *n*=4), AGK2 (1.109 ± 0.2717, *n*=4).  Ordinary one-way ANOVA (Tukey’s multiple comparisons test) F(2, 9) = 6.540, *p* =0.  Naïve vs Putrescine, *p =* 0.0448; Putrescine vs AGK2, *p =* 0.8813. |
|  | **GABA**  Naïve (0.3939 ± 0.06296, *n*=6), Putrescine (1.000 ± 0.000, *n*=6), AGK2 (0.693 ± 0.09777, *n*=6).  Ordinary one-way ANOVA (Tukey’s multiple comparisons test) 0176F(2, 15) = 20.82, *p* <0.0001  Naïve vs Putrescine, *p* <0.0001; Putrescine vs AGK2, *p =* 0.0031. |
| 3C | **Putrescine**  Scr (0.1614 ± 0.01457, *n*=9), Scr+Put (1.000 ± 0.000, *n*=9), shSIRT2+Put (0.9316 ± 0.1030, *n*=9).  Ordinary one-way ANOVA (Tukey’s multiple comparisons test) F(2, 24) = 60.08, *p* <0.0001  Scr vs Scr+Put, *p* <0.0001; Scr+Put vs shSIRT2+Put, *p =* 0.7033. |
|  | **N-Acetyl-GABA**  Scr (0.9651 ± 0.1697, *n*=6), Scr+Put (1.000 ± 0.000, *n*=6), shSIRT2+Put (1.692 ± 0.2120, *n*=6).  Ordinary one-way ANOVA (Tukey’s multiple comparisons test) F(2, 14) = 7.099, *p* =0.007  Scr vs Scr+Put, *p* =0.9868; Scr+Put vs shSIRT2+Put, *p =* 0.0155. |
|  | **GABA**  Scr (0.3851 ± 0.03424, *n*=9), Scr+Put (1.000 ± 0.000, *n*=9), shSIRT2+Put (0.8090 ± 0.04443, *n*=9).  Ordinary one-way ANOVA (Tukey’s multiple comparisons test) F(2, 24) = 94.43, *p* <0.0001  Scr vs Scr+Put, *p* <0.0001; Scr+Put vs shSIRT2+Put, *p =* 0.0010. |
| 3F | Vehicle (34.09 ± 4.014, *n*=19),Vehicle + Putrescine (98.79 ± 11.34, *n*=18), Putrescine + AGK2 (109.0 ± 12.01, *n*=19), Putrescine + AHK2 + KDS2010 (46.32 ± 6.726, *n*=15).  Ordinary one-way ANOVA (Tukey’s multiple comparisons test) F(3, 67) = 16.37, *p* <0.0001  Veh vs Veh+Put, *p* <0.0001; Veh vs Put+AGK2, *p*<0.0001; Veh vs Put+AGK2+KDS2010, *p* = 0.8046; Veh+Put vs Put+AGK2, *p =* 0.8599; Veh+Put vs Put+AGK2+KDS2010, *p* = 0.0017; Put+AGK2 vs Put+AGK2+KDS2010, *p* = 0.0001 |
| 4C | Vehicle (2.688 ± 0.8747, *n*=10), Veh+Put (19.25 ± 2.761, *n*=31), Put+DEAB (2.157 ± 0.5911, *n*=14), Put+AGK2 (3.941 ± 0.9385, *n*=18).  Ordinary one-way ANOVA (Dunnett’s multiple comparisons test) F(4, 86) = 7.878, *p* <0.0001  Veh vs Veh+Put, *p* = 0.0039; Veh+Put vs Put+DEAB, *p =* 0.0006; Veh+Put vs Put+AGK2, *p* = 0.0008. |
| 4D | Scr (32.88 ± 4.827, *n*=21), shALDH1A1 (10.70 ± 3.644, *n*=9), shSIRT2 (0.1470 ± 0.06544, *n*=11).  Ordinary one-way ANOVA (Holm-Sidak’s multiple comparisons test) F(2, 38) = 15.16, *p* <0.0001  Scr vs shALDH1A1, *p* = 0.0020; Scr vs shSIRT2, *p*<0.0001. |
| 5C | **Tonic GABA current**:  Scr (11.58 ± 0.9299, *n*=13), shALDH1A1 (6.655 ± 1.058, *n*=16), shSIRT2 (5.852 ± 0.7340, *n*=22)  Ordinary one-way ANOVA (Tukey’s multiple comparisons test) F(2, 48) = 10.59, *p* = 0.0002  Scr vs shALDH1A1, *p* = 0.0023; Scr vs shSIRT2, *p* = 0.0002; shALDH1A1 vs shSIRT2, *p* = 0.7862. |
|  | **sIPSC amplitude**:  Scr (43.8 ± 5.584, *n*=13), shALDH1A1 (45.96 ± 3.805, *n*=15), shSIRT2 (39.16 ± 2.870, *n*=21)  Ordinary one-way ANOVA (Tukey’s multiple comparisons test) F(2, 46) = 0.8819, *p* = 0.4209  Scr vs shALDH1A1, *p* = 0.9302; Scr vs shSIRT2, *p* = 0.6825; shALDH1A1 vs shSIRT2, *p* = 0.4137. |
|  | **sIPSC frequency**:  Scr (1.056 ± 0.1323, *n*=10), shALDH1A1 (0.9101 ± 0.1045, *n*=14), shSIRT2 (1.0004 ± 0.1227, *n*=21)  Ordinary one-way ANOVA (Tukey’s multiple comparisons test) F(2, 42) = 0.2892, *p* = 0.7503.  Scr vs shALDH1A1, *p* = 0.7494; Scr vs shSIRT2, *p* = 0.9575; shALDH1A1 vs shSIRT2, *p* = 0.8426. |
| 6B | **ALDH1A1 Intensity:**  WT (36.51 ± 1.235, *n*=30), APP/PS1 TG (43.14 ± 1.460, *n*=39).  Unpaired two tailed t-test; *p* = 0.0014 |
|  | **GFAP area x intensity:**  WT (4954 ± 543.5, *n*=30), APP/PS1 TG (12516 ± 1321, *n*=39).  Unpaired two tailed t-test; *p* <0.0001 |
| 6D | WT (3.076 ± 0.3260, *n*=33), APP/PS1 TG (4.254 ± 0.2659, *n*=68).  Unpaired two tailed t-test; *p* = 0.0093 |
| 6E | WT (0.07139 ± 0.02269, *n*=5), APP/PS1 TG (0.2808 ± 0.03390, *n*=6).  Unpaired two tailed t-test; *p* = 0.0008 |
| 6G | WT+Scr (69.14 ± 2.709, *n*=10), TG+Scr (57.40 ± 2.252, *n*=11), TG+shSIRT2 (55.56 ± 3.508, *n*=11)  Ordinary one-way ANOVA (Holm=Sidak’s multiple comparisons test) F(2, 29) = 4.801, *p* = 0.0158  WT+Scr vs TG+Scr, *p* = 0.0236; TG+Scr vs TG+shSIRT2, *p* = 0.0408; WT+Scr vs TG+shSIRT2, *p* = 0.6467. |
| 6I | **Tonic GABA current**:  WT+Scr (5.713 ± 0.9454, *n*=15), TG+Scr (10.34 ± 1.060, *n*=19), TG+shSIRT2 (5.908 ± 0.6065, *n*=13)  Ordinary one-way ANOVA (Tukey’s multiple comparisons test) F(2, 44) = 8.161, *p* = 0.0010  WT+Scr vs TG+Scr, *p* = 0.0027; TG+Scr vs TG+shSIRT2, *p* = 0.0060; WT+Scr vs TG+shSIRT2, *p* = 0.9899. |
|  | **sIPSC amplitude**:  WT+Scr (40.46 ± 2.445, *n*=11), TG+Scr (36.75 ± 2.597, *n*=20), TG+shSIRT2 (36.01 ± 3.416, *n*=14)  Ordinary one-way ANOVA (Tukey’s multiple comparisons test) F(2, 42) = 0.5428, *p* = 0.5852.  WT+Scr vs TG+Scr, *p* = 0.6593; TG+Scr vs TG+shSIRT2, *p* = 0.9804; WT+Scr vs TG+shSIRT2, *p* = 0.5944. |
|  | **sIPSC frequency**:  WT+Scr (1.103 ± 0.1030, *n*=12), TG+Scr (1.167 ± 0.1509, *n*=20), TG+shSIRT2 (0.9607 ± 0.1192, *n*=13)  Ordinary one-way ANOVA (Holm-Sidak’s multiple comparisons test) F(2, 42) = 0.5773, *p* = 0.5658.  WT+Scr vs TG+Scr, *p* = 0.7653; TG+Scr vs TG+shSIRT2, *p* = 0.6429; WT+Scr vs TG+shSIRT2, *p* = 0.7653. |
| 7A | Normal (6.853 ± 0.3111, *n*=8), AD (9.209 ± 0.6839, *n*=8)  Unpaired two-tailed t-test, *p* = 0.0073. |
| 7E | Normal (0.05290 ± 0.02155, *n*=4), AD (0.5285 ± 0.1576, *n*=4)  Unpaired two-tailed t-test, *p* = 0.0243. |
