# Supplementary material for: SIRT2 and ALDH1A1 as critical enzymes for astrocytic GABA production in Alzheimer’s disease
Source: Mol Neurodegener. 2025 Jan 15;20:6. doi: 10.1186/s13024-024-00788-8 (PMC11734448; doi:10.1186/s13024-024-00788-8)

SIRT2 and ALDH1A1 as critical enzymes for astrocytic GABA production in Alzheimer's Disease

Mridula Bhalla[1,2], Jinhyeong Joo[1,2], Daeun Kim[1,3], Jeong Im Shin[1,4], Yongmin Mason Park[1,2], Yeon Ha Ju[5], Uiyeol Park[5], Seonguk Yoo[6], Seung Jae Hyeon[5], Hyunbeom Lee[6], Junghee Lee[7,8]*, Hoon Ryu[5,9]*, C Justin Lee[1,2] *

Supplementary Figures

Figure S1 (related to Fig. 2) shRNA construct design and testing for shSIRT2

Figure S2 (related to Fig. 6) Astrocyte-specific knockdown of SIRT2 has partial effect on memory rescue in APP/PS1 mice.

Graphical abstract

Supplementary Figure 1

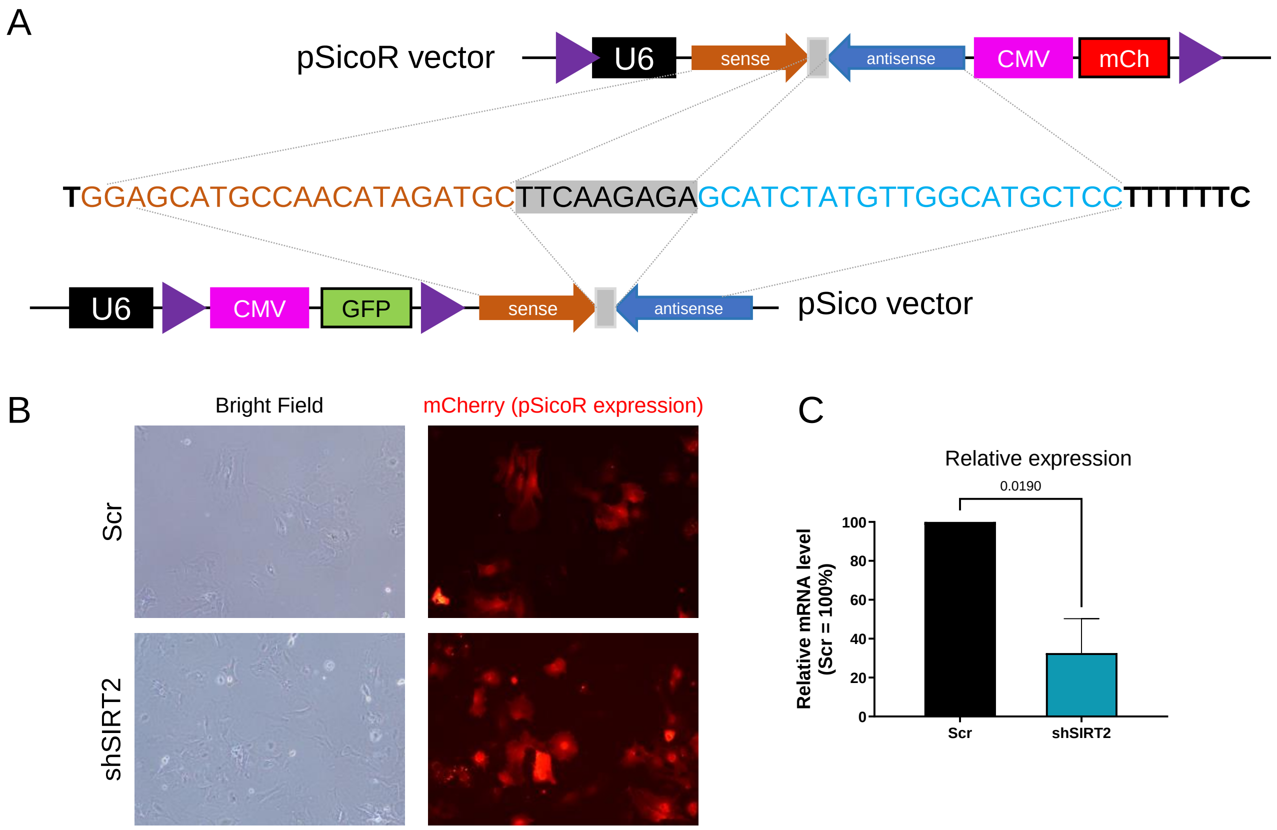

Figure S1. shRNA construct design and testing for shSIRT2

(A) Representative schematic for vectors pSico and pSicoR used in the study, indicating location of shRNA sequence; (Middle) shSIRT2 sequence used in this study.

(B) Representative images of primary hippocampal astrocyte cultures microporated with pSicoR-Scr-mCherry (left) and pSicoR-shSIRT2-mCherry (right).

(C) Representative bar graph from qRT-PCR performed using RNA isolated from microporated primary astrocytes (value above bars represents p-value from Unpaired t-test).

Supplementary Figure 2

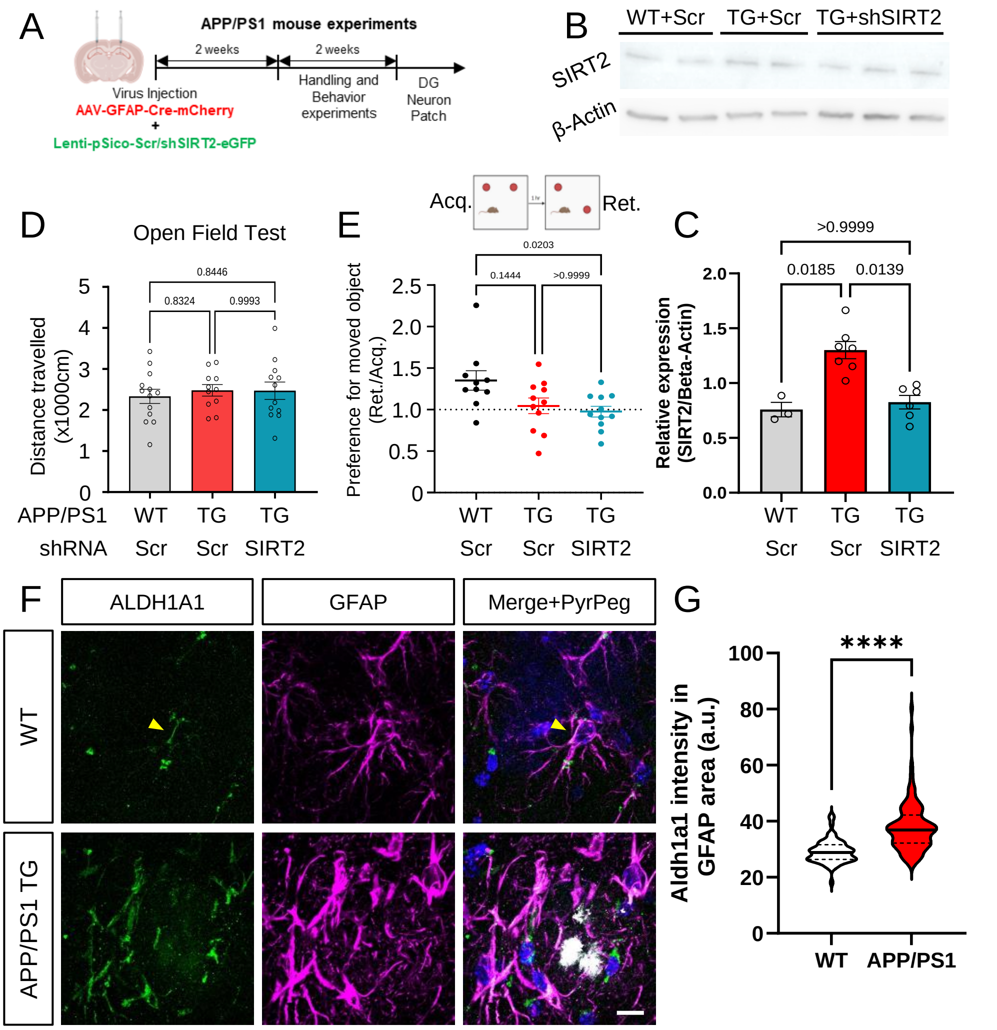

Figure S2. Astrocyte-specific knockdown of SIRT2 has partial effect on memory rescue in APP/PS1 mice.

(A) Schematic for APP/PS1 animal experiments

(B) Representative western blots used for quantification of SIRT2 KD in virus injected hippocampi.

(C) Bar graph for western blot analysis for SIRT2 knockdown in Scr- and shSIRT2-injected APP/PS1 animal hippocampus (data obtained from 2 separate experiments, N=3-7 each group)

(D) Representative bar graph for Open Field Test, indicating distance travelled by the animals.

(E) Schematic for Novel Place Recognition experiment (top); Representative dot-plot for the ratio of preference for the moved object shown by the animals (Retention/Acquisition; bottom).

(F) Images representing ALDH1A1 and GFAP immunoreactivity around Aβ plaques in the CA1 *stratum radiatum* in the hippocampus of APP/PS1 mice (Scale bar 10μm).

(G) Violin plot representing the astrocytic ALDH1A1 intensity from images in **(F)** (N=3, Mann-Whitney test).

Data represents Median ± quartiles or Mean ± SEM. Data points indicate individual animals. Values above graphs refer to p values from One way ANOVA Kruskal-Wallis test.

Graphical abstract

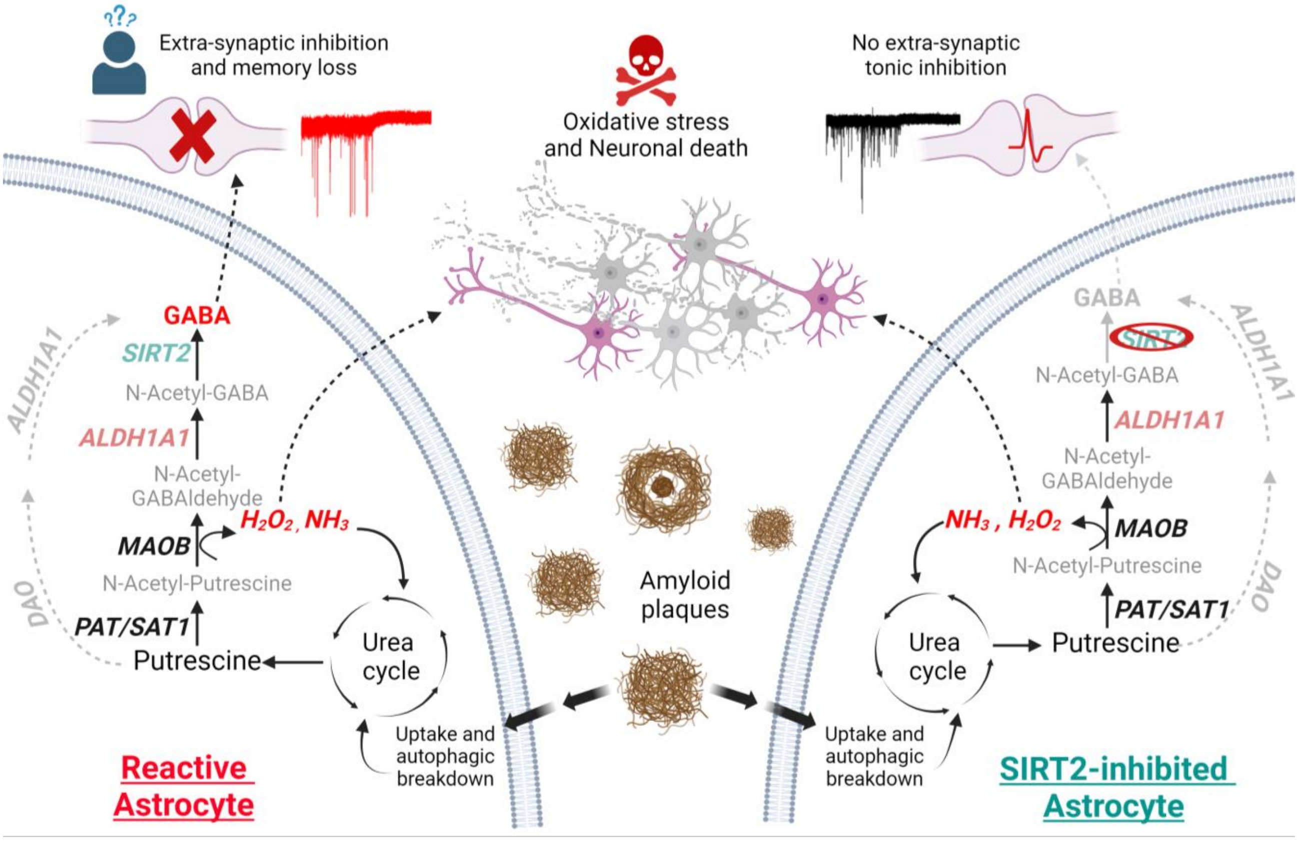

Supplement: Supplementary file 2 — Supplementary Material 2: Figure S1: shRNA construct design and testing for shSIRT2. Figure S2: Astrocyte-specific knockdown of SIRT2 has partial effect on memory rescue in APP/PS1 mice. [file 13024_2024_788_MOESM2_ESM.pdf]
